# Cancer‐Associated Fibroblasts: Heterogeneity, Cancer Pathogenesis, and Therapeutic Targets

**DOI:** 10.1002/mco2.70292

**Published:** 2025-07-11

**Authors:** Yutao Li, Qingyun Liu, Xilin Jing, Yuqi Wang, Xiaohua Jia, Xing Yang, Kezhong Chen

**Affiliations:** ^1^ Department of Thoracic Surgery Peking University People's Hospital Beijing China; ^2^ Thoracic Oncology Institute Peking University People's Hospital Beijing China; ^3^ Research Unit of Intelligence Diagnosis and Treatment in Early Non‐Small Cell Lung Cancer Chinese Academy of Medical Sciences 2021RU002 Peking University People's Hospital Beijing China; ^4^ Peking University Health Science Center Beijing China; ^5^ Department of Nuclear Medicine Peking University People's Hospital Beijing China; ^6^ Key Laboratory of Molecular Imaging of Chinese Academy of Sciences Institute of Automation Chinese Academy of Sciences Beijing China

**Keywords:** cancer‐associated fibroblasts, heterogeneity, pathogenesis, theranostic, tumor microenvironment

## Abstract

Cancer‐associated fibroblasts (CAFs) are functionally diverse stromal regulators that orchestrate tumor progression, metastasis, and therapy resistance through dynamic crosstalk within the tumor microenvironment (TME). Recent advances in single‐cell multiomics and spatial transcriptomics have identified conserved CAF subtypes with distinct molecular signatures, spatial distributions, and context‐dependent roles, highlighting their dual capacity to promote immunosuppression or restrain tumor growth. However, therapeutic strategies struggle to reconcile this functional duality, hindering clinical translation. This review systematically categorizes CAF subtypes by origin, biomarkers, and TME‐specific functions, focusing on their roles in chemoresistance, maintenance of stemness, and formation of immunosuppressive niches. We evaluate emerging targeting approaches, including selective depletion of tumor‐promoting subsets (e.g., fibroblast activation protein+ CAFs), epigenetic reprogramming toward antitumor phenotypes, and inhibition of CXCL12/CXCR4 or transforming growth factor‐beta signaling pathways. Spatial multiomics‐driven combinatorial therapies, such as the synergistic use of CAFs and immune checkpoint inhibitors, are highlighted as strategies to overcome microenvironment‐driven resistance. By integrating CAF biology with translational advances, this work provides a roadmap for developing subtype‐specific biomarkers and precision stromal therapies, directly informing efforts to disrupt tumor‐stroma coevolution. Key concepts include spatial transcriptomics, stromal reprogramming, and tumor‐stroma coevolution, offering actionable insights for both mechanistic research and clinical innovation.

## Graphical abstract

Cancer‐associated fibroblasts (CAFs) arise from normal fibroblasts in response to various tumor microenvironmental signals, including cytokines such as TGF‐β and IL‐6, paracrine factors like PDGFs, Notch signaling, extracellular vesicles, as well as aging, inflammation, hypoxia, oxidative stress, and increased extracellular matrix (ECM) stiffness. Upon activation, these fibroblasts differentiate into distinct subtypes—myofibroblastic CAFs (myCAFs), inflammatory CAFs (iCAFs), and antigen‐presenting CAFs (apCAFs)—each contributing uniquely to tumor progression. CAFs remodel the ECM and secrete inflammatory cytokines such as IL‐1, IL‐6, and CXCL12, fostering an immunosuppressive environment by modulating immune cell populations. Through MHC‐II‐mediated interactions with CD4+ T cells and the recruitment or activation of immunosuppressive cells including MDSCs, Tregs, Th2 cells, ILC2s, NK2 cells, NKT2 cells, and M2 macrophages, CAFs blunt antitumor immunity. Collectively, these activities promote cancer cell proliferation, angiogenesis, epithelial‐to‐mesenchymal transition (EMT), and metastasis, positioning CAFs as central orchestrators of tumor development and immune evasion (created with www.biorender.com).

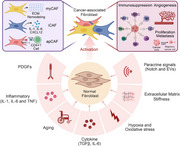

## 1 Introduction

Cancer remains a leading cause of mortality worldwide, with incidence and death rates continuing to rise. In 2024, the United States alone is projected to see 2,001,140 new cancer cases and 611,720 cancer‐related deaths [1]. Cancer‐associated fibroblasts (CAFs) are a heterogeneous population of activated stromal cells that constitute a major component of the tumor microenvironment (TME). Unlike normal fibroblasts, CAFs adopt a protumorigenic phenotype characterized by enhanced proliferation, migration, and extracellular matrix (ECM) remodeling [2]. They originate from diverse sources, including resident fibroblasts, bone marrow‐derived mesenchymal stem cells (MSCs), pericytes, and endothelial cells undergoing EndMT, and are activated by cytokines such as transforming growth factor‐beta (TGF‐β), platelet‐derived growth factor (PDGF), and inflammatory signals [3, 4, 5]. Once activated, CAFs secrete various bioactive molecules, including growth factors (e.g., vascular endothelial growth factor [VEGF], hepatocyte growth factor [HGF]), chemokines (e.g., CXCL12, CXCL5), and ECM proteins, thereby promoting tumor cell survival, angiogenesis, and immune evasion [6]. Additionally, CAFs engage in metabolic crosstalk with cancer cells, supplying metabolites such as acetate and lactate to fuel tumor growth and enhance adaptation to hypoxia [7].

Several key markers are commonly used to identify CAFs, including alpha‐smooth muscle actin (α‐SMA), fibroblast activation protein (FAP), and PDGF receptors (PDGFR‐α/β) [8, 9]. However, due to the heterogeneity of CAFs, no single marker universally defines all CAF subsets. This diversity suggests that distinct CAF subpopulations perform specialized functions within the TME, actively contributing to tumor progression. While most CAFs exhibit tumor‐promoting functions, certain subsets have been reported to exert tumor‐suppressive effects, emphasizing the complexity of CAF biology and the need for further research to elucidate their context‐dependent roles in cancer development and therapy resistance [10]. Recent advances in single‐cell transcriptomics and lineage tracing techniques have revealed significant CAF heterogeneity, leading to the classification of distinct CAF subtypes based on molecular signatures and functional attributes. Broadly, CAFs can be categorized into myofibroblastic CAFs (myCAFs), inflammatory CAFs (iCAFs), and antigen‐presenting CAFs (apCAFs) [11]. MyCAFs, characterized by high α‐SMA expression and a contractile phenotype, primarily contribute to ECM remodeling and fibrosis [12]. In contrast, iCAFs exhibit a secretory phenotype, producing high levels of inflammatory cytokines such as interleukin‐6 (IL‐6) and IL‐8, thereby modulating immune cell recruitment and tumor‐associated inflammation [13, 14]. A recently identified subset, apCAFs, expresses major histocompatibility complex (MHC) class II molecules and has been implicated in antigen presentation, potentially influencing antitumor immune responses, although their precise role remains under investigation [15]. Additional CAF subtypes with distinct metabolic and signaling properties continue to be identified, highlighting the dynamic and context‐dependent nature of CAF functions across different tumor types [16].

A critical aspect of CAF biology is their role in facilitating cancer progression and therapy resistance. CAFs promote tumor invasion by remodeling the ECM through MMP‐mediated degradation and collagen crosslinking, thereby creating a fibrotic niche [17, 18]. Additionally, CAFs produce a range of protumorigenic factors, including TGF‐β, HGF, and fibroblast growth factors (FGFs), that support cancer cell survival, proliferation, and epithelial‐to‐mesenchymal transition (EMT) [19, 20]. Moreover, CAFs modulate immune responses by recruiting immunosuppressive cells such as regulatory T cells (Treg cells) and myeloid‐derived suppressor cells (MDSCs) while simultaneously impairing cytotoxic T lymphocyte (CTL) function, thereby fostering an immune‐evasive TME [21]. CAFs also contribute to therapeutic resistance by altering drug penetration, promoting metabolic reprogramming, and activating survival pathways in tumor cells, ultimately reducing the efficacy of chemotherapy, radiotherapy, and targeted therapies [22, 23, 24, 25]. Given their pivotal role in tumor biology, CAFs have emerged as promising therapeutic targets [26]. Strategies to modulate CAF activity include targeting CAF‐specific signaling pathways, reprogramming tumor‐promoting CAFs into tumor‐suppressive phenotypes, and disrupting CAF–cancer cell interactions [27]. However, therapeutic interventions must consider CAF heterogeneity to avoid unintended consequences, as some CAF subsets may have tumor‐restraining effects [28]. A deeper understanding of CAF plasticity and their dynamic interactions within the TME is essential for developing precision therapeutics aimed at mitigating CAF‐mediated tumor progression and treatment resistance.

Although numerous reviews in recent years have examined the heterogeneity, pathogenic mechanisms, and therapeutic strategies related to CAFs, there is still a lack of a comprehensive review that systematically integrates these aspects while bridging laboratory discoveries with clinical applications from a translational perspective [29, 30, 31, 32]. Additionally, the emerging concept of theranostics, stemming from advances in imaging technologies and therapeutic strategies targeting CAFs, has yet to be thoroughly reviewed. Theranostics, which combines diagnostic imaging with targeted therapy, holds great promise for advancing precision oncology [33, 34, 35]. We propose that CAFs are a compelling target for theranostic approaches, and that their effective clinical translation depends on a thorough understanding of their heterogeneity and functional roles in the TME. Therefore, a systematic review that integrates these elements is both timely and necessary to guide future research and clinical development.

This review begins by exploring the heterogeneity of CAFs across different tumor types and among subtypes within the same tumor type, as reported in recent studies. We then summarize the functional roles of different CAF subsets in the TME, emphasizing their involvement in immune modulation, stromal remodeling, angiogenesis, and the promotion of EMT. Drawing on these functional insights and related molecular markers, we also review positron emission tomography (PET) and fluorescence imaging modalities developed to target CAFs. Furthermore, we provided a comprehensive overview of therapeutic strategies aimed at CAFs, including targeting CAF‐related molecular pathways, selectively depleting specific phenotypic subpopulations, and reprogramming CAFs into quiescent states. Finally, from a clinical translational perspective, we discussed the challenges and potential solutions associated with imaging and therapeutic interventions targeting CAFs and introduced the concept of CAF‐targeted theranostics as a promising strategy for precision cancer therapy.

## 2 Heterogeneity of CAFs

The spatial and temporal heterogeneity of CAFs underlies their multifaceted roles in remodeling the TME. Spatial organization determines niche‐specific functions, such as ECM remodeling and angiogenesis, while temporal plasticity (driven by reversible fibroblast dedifferentiation in response to tumor‐derived cues) enables adaptive transitions between activation states. This functional versatility, rooted in diverse tissue origins and molecular drivers, amplifies CAF heterogeneity and their context‐dependent contributions to tumor progression. Deciphering these dynamics requires integrating single‐cell multiomics, spatial imaging, and functional assays to map the molecular determinants of CAF plasticity and subtype‐specific TME interactions. Such insights are critical for developing precision therapies that disrupt the CAF–TME axis by targeting dedifferentiation pathways or spatially restricted functional specializations, ultimately harnessing CAF heterogeneity as a therapeutic vulnerability.

### 2.1 Cellular Origins of CAFs

#### 2.1.1 MSCs as Precursors of CAFs

MSCs serve as a primary source of CAFs within the TME. These multipotent stromal cells, which originate from bone marrow, adipose tissue, and other mesenchymal tissues, can be actively recruited to tumor sites by cytokines and chemokines, including TGF‐β, PDGF, and stromal cell‐derived factor‐1 [36]. Upon exposure to these tumor‐derived signals, MSCs differentiate into CAFs, acquiring specific characteristics, such as elevated expression levels of α‐SMA, FAP, and vimentin [37].

The differentiation of MSCs into CAFs is predominantly driven by the TGF‐β/SMAD, NF‐κB, and JAK/STAT signaling pathways. Among these, TGF‐β plays a particularly critical role by initiating a transcriptional program that enhances the expression of CAF‐associated markers and reinforces their profibrotic [38]. The activation of the NF‐κB and STAT3 pathways further amplifies the protumorigenic functions of MSC‐derived CAFs, leading to increased cytokine production and therapeutic resistance [39, 40]. Additionally, MSCs can undergo functional differentiation regulated by factors such as myocardin‐related transcription factor A, which stabilizes their tumor‐promoting CAF phenotype [37]. Notably, MSC‐derived CAFs exhibit a high degree of plasticity, contributing to their heterogeneity within tumors.

MSC‐derived CAFs have been identified in various cancer types, including breast, lung, and pancreatic cancers, highlighting their significant role in tumor progression [37, 41, 42]. However, the precise molecular mechanisms governing the recruitment and differentiation of MSCs into CAFs remain incompletely understood, particularly regarding the heterogeneity of CAF subsets and their context‐dependent interactions with immune cells. Elucidating these pathways is critical for a more comprehensive understanding of the contributions of MSCs to CAF heterogeneity and their functional diversity across different tumor contexts [43].

#### 2.1.2 EndMT in CAF Formation

In addition to MSCs, endothelial cells represent another significant source of CAFs through a process known as EndMT [44]. EndMT involves a phenotypic conversion in which endothelial cells lose their characteristic markers, such as CD31 and VE‐cadherin, while acquiring mesenchymal traits, including the expression of α‐SMA and FAP [45, 46]. This transition facilitates the detachment of endothelial cells from the vascular endothelium, allowing them to integrate into the tumor stroma as functionally active CAFs.

EndMT in the formation of CAFs is primarily driven by key tumor‐derived factors, particularly TGF‐β and IL‐6 [47]. Among these factors, TGF‐β plays a central role by activating the SMAD‐dependent signaling cascade and interacting with other pathways, such as Notch and Wnt/β‐catenin, thereby reinforcing the mesenchymal phenotype [48, 49]. Additionally, hypoxia, a hallmark of the TME, promotes EndMT by upregulating hypoxia‐inducible factor‐1α (HIF‐1α), which enhances the expression of transcription factors that induce EndMT, including Snail, Slug, and Twist [50, 51].

Notably, EndMT‐generated CAFs exhibit significant plasticity, enabling them to dynamically respond to environmental cues [52]. Although the extent of EndMT's contribution to the CAF population varies across different cancer types, it represents a crucial alternative mechanism for fibroblast activation within tumors. Further investigation is necessary to delineate the regulatory networks that govern EndMT and its role in shaping the tumor stroma.

#### 2.1.3 Fibroblast Plasticity and Dedifferentiation in the TME

Beyond the recruitment and transdifferentiation of MSCs and endothelial cells, CAFs can also originate from the phenotypic plasticity and dedifferentiation of resident fibroblasts within the TME [53]. Traditionally considered terminally differentiated, fibroblasts have been shown to undergo dynamic reprogramming in response to tumor‐derived signals, shifting among quiescent, activated, and CAF‐like states [54]. This plasticity enables fibroblasts to adapt to the evolving TME and contributes to CAF heterogeneity.

Key regulators of fibroblast plasticity include TGF‐β, PDGF, and FGF, which activate signaling pathways such as TGF‐β/SMAD, JAK/STAT, and Wnt/β‐catenin [55]. Under persistent stimulation, fibroblasts can lose their homeostatic identity and adopt a more primitive, mesenchymal‐like state. This transition is characterized by the downregulation of quiescence markers, such as caveolin‐1, and the upregulation of CAF markers, including α‐SMA and FAP [56, 57]. Additionally, epigenetic modifications, including DNA methylation and histone remodeling, play a crucial role in stabilizing the CAF phenotype [58, 59].

Importantly, fibroblast dedifferentiation may serve as a reversible mechanism, allowing cells to transition between different activation states in response to tumor‐derived cues [60]. This plasticity not only expands the potential sources of CAFs but also reinforces their heterogeneity, as fibroblasts from diverse tissue origins may respond differently to oncogenic signals [61]. Further investigation into the molecular drivers of fibroblast plasticity and dedifferentiation is essential for understanding CAF diversity and its impact on tumor progression.

### 2.2 Molecular and Functional Diversity of CAFs

#### 2.2.1 CAF Subtypes: MyCAFs, Senescent CAFs, and Activated CAFs

CAFs exhibit profound functional and molecular heterogeneity, with distinct subtypes contributing to tumor progression through various mechanisms. MyCAFs, characterized by the expression of αSMA, play a central role in ECM remodeling and tissue contractility. In pancreatic ductal adenocarcinoma (PDAC), myCAFs promote immunosuppression by recruiting Treg cells and expressing programmed death‐ligand 1 (PD‐L1). In contrast, in breast cancer, they enhance tumor invasion by secreting CXCL12 and facilitating collagen contraction [62, 63]. Notably, epidermal growth factor receptor (EGFR)/ERBB2 signaling in PDAC myCAFs drives metastasis through an autocrine loop involving amphiregulin, highlighting their therapeutic vulnerability [12].

senCAFs, characterized by a senescence‐associated secretory phenotype (SASP), secrete proinflammatory cytokines and ECM components that suppress antitumor immunity. In breast cancer, senCAFs inhibit natural killer (NK) cell activity through collagen deposition, which correlates with tumor recurrence [64]. In PDAC, p16⁺ senCAFs localized near ductal structures promote immunosuppression and chemoresistance, effects that can be alleviated by senolytic agents [65]. Additionally, hypoxia exacerbates their SASP in esophageal cancer, driving cancer stemness through the secretion of insulin‐like growth factor 1 (IGF1) [66].

Activated CAFs, induced by TGF‐β or inflammatory cytokines, exhibit protumorigenic phenotypes. In colon cancer, these cells reprogram the TME through the induction of EMT and glycolytic remodeling, thereby fostering immunosuppression [67]. In gastric cancer, activated CAFs secrete paracrine factors that enhance resistance to 5‐fluorouracil [68]. Additionally, Snail1⁺ fibroblasts in colon cancer stimulate tumor cell migration via MCP‐3‐mediated signaling [69].

**FIGURE 1:**
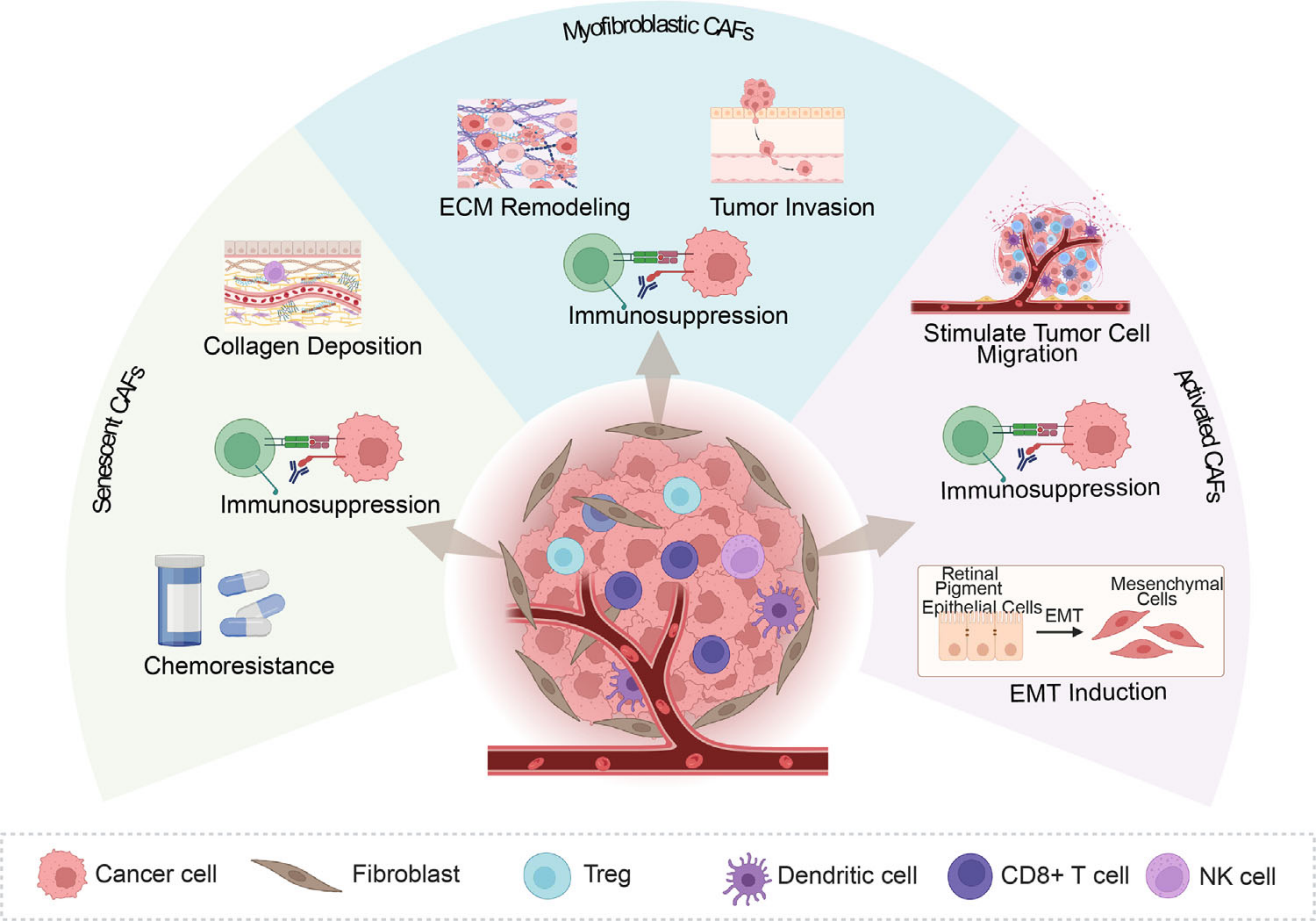
Classification of cancer‐associated fibroblasts (CAFs) and their diverse functions. CAFs can be categorized into myofibroblastic CAFs, senescent CAFs, and activated CAFs, each influencing the tumor microenvironment and cancer progression through distinct mechanisms. *Abbreviations*: CAFs, cancer‐associated fibroblasts; ECM, extracellular Matrix; EMT, epithelial–mesenchymal transition (created in https://BioRender.com).

Interactions among CAF subtypes are dynamic and context dependent. For example, hypoxia can reprogram myCAFs into senCAFs, thereby amplifying immunosuppression. Targeting these subtypes offers therapeutic opportunities: senolytic agents can delay tumor growth by eliminating senCAFs, while inhibition of the EGFR preferentially suppresses metastatic myCAFs in PDAC. Additionally, spatial transcriptomics has shown that POSTN⁺ myCAFs in non‐small‐cell lung cancer (NSCLC) correlate with resistance to immunotherapy [70]. A deeper understanding of CAF heterogeneity and plasticity will be crucial for refining therapeutic strategies and improving clinical outcomes in cancer treatment (Figure 1).

#### 2.2.2 Molecular Signatures of CAFs: Transcriptomic and Proteomic Profiling

The molecular heterogeneity of CAFs has been systematically dissected through transcriptomic and proteomic profiling, revealing distinct functional subtypes and regulatory mechanisms. Single‐cell RNA sequencing (scRNA‐seq) has emerged as a cornerstone technology for classifying CAFs into transcriptionally distinct subsets. For instance, pan‐cancer analyses have identified four major subtypes: iCAFs, matrix CAFs (mCAFs), proliferative CAFs, and metabolic CAFs, each characterized by unique gene expression profiles. iCAFs are enriched in chemokines such as CCL11 and CXCL12, which mediate immune cell recruitment, whereas mCAFs exhibit high expression of ECM remodeling genes, including MMP11 and COL1A1, reflecting their role in tissue remodeling [71]. Notably, cancer type‐specific CAF subtypes have been reported, such as vascular CAFs, marked by NOTCH3 and COL18A1 in breast cancer, and tumor‐associated CAFs (tCAFs), enriched in MME and NDRG1 in pancreatic cancer [72].

The functional specialization of CAFs is further elucidated by lineage‐tracing studies. For instance, macrophage–myofibroblast transition, driven by Smad3 signaling, generates CAFs with protumorigenic properties in NSCLC [73]. In gastric cancer, scRNA‐seq has identified ECM CAFs that promote tumor invasion and correlate with poor prognosis. Similarly, glioblastoma CAFs have been shown to enhance stemness and angiogenesis through growth factor secretion and ECM remodeling [74].

Proteomic profiling complements transcriptomics by validating functional proteins and revealing spatial dynamics. For instance, periostin and CD36 have been identified as markers of CAFs in ovarian cancer, with their spatial distribution correlating with patient survival [75]. In pancreatic cancer, the LAMA5/ITGA4 axis drives acinar‐to‐ductal metaplasia, a precursor to carcinogenesis, through STAT3 activation [76]. Additionally, proteomics has uncovered NNMT as a master metabolic regulator in ovarian CAFs, mediating DNA methylation and contributing to drug resistance [59]. The integration of multiomics has provided deeper insights into CAF biology. A cross‐species study identified three conserved CAF phenotypes: steady‐state‐like, mechanoresponsive, and immunomodulatory, highlighting their evolutionary conservation and therapeutic vulnerability [77]. In glioblastoma, fibronectin (FN1) has been validated as a key CAF‐secreted factor that promotes mesenchymal transition and invasion [78].

Clinically, CAF‐derived molecular signatures show promise as biomarkers. For instance, collagen XII has been shown to predict metastatic relapse in breast cancer [79], while APOE–LRP5 signaling correlates with poor outcomes in ovarian cancer [75]. Targeting CAF‐specific pathways is emerging as a promising therapeutic strategy. The molecular heterogeneity of CAFs underscores their dynamic role in tumor progression. Although technical limitations and functional plasticity pose challenges, these findings highlight the necessity of integrating multiomics with spatial and functional analyses to fully elucidate CAF‐mediated mechanisms. Such efforts will be critical for translating CAF heterogeneity into precision oncology.

#### 2.2.3 Functional Roles in ECM Remodeling, Growth Factor Secretion, and Angiogenesis

CAFs exhibit remarkable molecular and functional diversity, leading to tumor‐specific contributions in ECM remodeling, growth factor signaling, and angiogenesis [80]. These processes vary among CAF subpopulations and are influenced by cancer types, genetic mutations, and microenvironmental cues.

CAFs dynamically restructure the ECM through collagen deposition, degradation, and crosslinking, creating a mechanically and biochemically heterogeneous niche. For instance, in colorectal cancer (CRC) liver metastases, portal‐derived CAFs adopt a contractile myofibroblastic phenotype and produce collagen IV to promote desmoplasia. This stromal stiffening paradoxically restricts cancer cell proliferation while enhancing metastatic colonization by inducing integrin‐mediated signaling [81]. Conversely, in oral squamous cell carcinoma (OSCC), CAFs secrete small extracellular vesicles (sEVs) enriched in lysyl oxidase (LOX), which triggers collagen crosslinking and EMT via the p‐focal adhesion kinase (FAK)/p‐paxillin/YAP axis. Notably, LOX‐positive sEVs are preferentially secreted by αSMA‐negative CAFs, highlighting subpopulation‐specific ECM‐modifying strategies [82, 83]. Matrix metalloproteinases (MMPs) and tissue inhibitors of metalloproteinases (TIMPs) further emphasize CAF heterogeneity. In hepatocellular carcinoma (HCC), CAFs adjacent to tumors express MMP‐9 and TIMP‐1 in a spatially restricted manner, promoting ECM degradation at the invasion front while preserving structural integrity elsewhere [84, 85]. This dual role is mirrored in Ewing sarcoma, where CAF‐like cells secrete protumorigenic ECM proteins, such as periostin, to support metastasis while simultaneously expressing TIMP‐2 to limit excessive ECM breakdown [86]. Such functional specialization ensures precise ECM remodeling tailored to the stages of tumor progression.

CAFs secrete a diverse array of growth factors, cytokines, and chemokines that modulate tumor–immune crosstalk and metabolic reprogramming. However, their secretory profiles are not generic; rather, they align with specific CAF subtypes. For instance, TGF‐β secreted by CAFs in HCC and CRC drives EMT and immunosuppression, yet its efficacy depends on CAF polarization: N2‐polarized CAFs enhance TGF‐β signaling to recruit immunosuppressive neutrophils, whereas M2‐like CAFs prioritize the paracrine activation of PD‐L1 expression on tumor cells [81, 84, 87, 88]. Similarly, FGF19 secretion in CRC liver metastases is restricted to inflammatory CAFs (CD14+), which activate the FGFR4–JAK2–STAT3 pathway to promote neutrophil extracellular trap formation. This process creates a nutrient‐rich microenvironment by degrading extracellular DNA, in contrast to αSMA^+^ CAFs that primarily sustain fibrotic stroma [88, 89]. Notably, IGF2 secretion by CAFs in pancreatic cancer is associated with EMT‐related CAFs, which upregulate PD‐L1 to evade T‐cell surveillance [6]. These examples illustrate how distinct CAF subpopulations encode specific growth factor signatures to orchestrate tumor progression.

CAF‐driven angiogenesis is highly heterogeneous, with subpopulations employing distinct molecular and metabolic strategies to remodel the vasculature. In colon cancer, CAFs adjacent to oncogenic KRAS‐mutant tumors secrete VEGF A (VEGFA) and HIF‐1α, promoting endothelial sprouting through mTORC2‐AKT activation. However, VEGFA expression is selectively upregulated in SULF1‐negative CAFs, which lack heparan sulfate proteoglycan modification, thereby increasing VEGFA bioavailability [90, 91]. Conversely, in esophageal squamous cell carcinoma, CAF‐derived milk fat globule–EGF factor 8 (MFGE8) binds to integrin receptors on endothelial cells, activating the PI3K/AKT/ERK pathways to enhance vascular permeability. This effect is amplified by lipid metabolism reprogramming in CAFs, which accumulate lipids to provide energy for endothelial proliferation [92]. Epigenetic regulation further diversifies CAF angiogenic functions. FTO‐mediated m6A demethylation in conjunctival melanoma CAFs stabilizes VEGFA and EGR1 transcripts, whereas gut microbiota‐derived butyrate suppresses angiogenesis by inhibiting histone deacetylase 3 and reducing SULF1 expression in CRC CAFs [91, 93]. The PDPN/CCL2/STAT3 feedback loop in CAFs sustains proangiogenic polarization in CRC; however, this is disrupted in LRRC15+ immunosuppressive CAFs, which instead secrete angiostatic factors such as TIMP‐3 [94, 95]. Such contextual angiogenic programs underscore CAF heterogeneity as a critical determinant of vascular remodeling.

In summary, CAF heterogeneity enables specialized contributions to ECM remodeling, growth factor signaling, and angiogenesis, with each role tailored to specific tumor contexts. Unraveling the molecular determinants of these functional specializations will aid in developing precision therapies that disrupt the CAF–TME axis in a subtype‐specific manner.

### 2.3 Spatial and Temporal Heterogeneity in TMEs

CAFs exhibit profound spatial and temporal heterogeneity, which greatly influences tumor progression, immune modulation, and therapeutic responses. This duality reflects their adaptive responses to microenvironmental cues and therapeutic interventions.

CAFs display distinct spatial distributions across various tumor types and microanatomical niches. In NSCLC, imaging mass cytometry identified four CAF subpopulations: hypoxia‐associated tCAFs, which promote immune exhaustion, and inflammatory iCAFs/ifnCAFs, which are enriched in inflamed regions, highlighting their spatially segregated functions [96]. Similarly, in breast cancer, FAP‐positive (FAP+) CAFs demonstrated peritumoral and perivascular localization, with dipeptidyl peptidase 4 (DPP4)/YAP1‐driven plasticity linking their distribution to immune exclusion and invasion [97]. In CRC, T cell‐infiltrating CAFs expressing NECTIN2/CD40 localized adjacent to T cells, suppressing their activation through direct signaling [98]. These findings underscore how spatial positioning endows CAFs with context‐dependent roles in immune regulation and tissue remodeling.

CAFs undergo dynamic changes over time, driven by tumor progression and therapeutic interventions. In high‐grade serous ovarian cancer, chemotherapy reshapes CAF populations by reducing the presence of ECM–myCAFs while increasing iCAFs, which alters CD8+ T cell dynamics and promotes immune evasion [99]. Mouse models of triple‐negative breast cancer (TNBC) revealed temporal shifts in CAF subpopulations, with PDGFRβ+ CAFs dominating established tumors compared with PDGFRα+ CAFs in healthy tissue [100]. In NSCLC, specific CAF subsets such as CAF7 (PDGFRA−/PDGFRB+/FAP+) were correlated with poor prognosis and immune suppression, while CAF13 (PDGFRA+/PDGFRB+/FAP−) was associated with favorable outcomes in EGFR‐mutant tumors [101]. These temporal dynamics highlight the adaptability of CAFs to evolving microenvironments.

Spatial and temporal heterogeneity of CAFs is governed by signaling pathways and microenvironmental interactions. Hypoxia drives glycolysis and ECM remodeling in tCAFs in NSCLC [96], while YAP1 activation mediates FAP‐positive (FAP+) CAF plasticity in HCC [97]. Therapeutically, targeting YAP1 or DPP4 disrupts immunosuppressive CAF phenotypes, enhancing T cell infiltration [97, 99]. In head and neck squamous cell carcinoma, CCL19‐positive fibroblast‐like CAFs within tertiary lymphoid structures correlate with immunotherapy response, suggesting niche‐specific therapeutic targeting [102].

The spatial and temporal heterogeneity of CAFs reflects their multifaceted roles in TME remodeling. While spatial distribution determines niche‐specific functions, temporal dynamics allow for adaptive responses to therapy. Integrating single‐cell multiomics, spatial imaging, and functional assays will be essential for deciphering the complexity of CAF heterogeneity and harnessing its therapeutic potential.

## 3 Mechanisms of CAFs in Cancer Pathogenesis

CAFs in the TME exist in multiple subtypes and play a critical role in tumor initiation, progression, and drug resistance. They contribute to the activation of key signaling pathways, including TGF‐β and Wnt, and interact with cancer cells through mechanisms such as ECM remodeling, maintenance of cancer cell stemness, and immune regulation. These interactions form a complex network that influences tumor dynamics. A comprehensive analysis of these networks can provide deeper insights into the role of CAFs in tumor progression and aid in identifying novel and precise therapeutic targets, ultimately enhancing the efficacy of antitumor treatments.

### 3.1 Immune Suppressive Microenvironment

Numerous studies on various solid tumors have demonstrated that the immunosuppressive microenvironment, consisting of immunosuppressive cells, ECM, and other tumor‐associated components, plays a critical role in tumor initiation and progression [103, 104, 105, 106, 107]. As a key component of the TME, CAFs contribute to the formation of an immunosuppressive microenvironment through matrix remodeling, cytokine secretion, and direct inhibition of CTLs [80, 108, 109, 110]. Reshaping the immunosuppressive TME and enhancing the body's intrinsic antitumor response are central objectives in contemporary cancer treatment research [111, 112, 113]. The molecular mechanisms underlying CAF function in the immunosuppressive microenvironment have been extensively studied, involving multiple pathways such as TGF‐β, Wnt, MAPK, and NF‐kappaB [24, 114, 115]. Rather than focusing on a single pathway, this study adopts a broader perspective, examining the specific roles of CAFs to better align with diagnostic and therapeutic strategies targeting these mechanisms.

#### 3.1.1 Expressing Immune Checkpoint Ligands

Cancer evades immune surveillance through various mechanisms, with the PD‐1/PD‐L1 signaling pathway playing a central role by inducing apoptosis in antigen‐specific T cells while inhibiting apoptosis in Treg cells [116, 117, 118, 119]. Consequently, numerous immunotherapeutic strategies targeting PD‐1 and PD‐L1 have been developed, leading to significant advancements in cancer treatment [120, 121]. Similar to PD‐1/PD‐L1, CTLA‐4 is a crucial molecule in maintaining an immunosuppressive environment and is expressed in various tumors. Primarily found on T cells, CTLA‐4 competes with CD28 for binding to CD80/CD86, thereby blocking the costimulatory signals necessary for T cell proliferation during the initiation phase of the immune response [122]. As the first approved immune checkpoint inhibitor (ICI), the humanized CTLA‐4 antibody ipilimumab has transformed clinical cancer treatment, significantly improving the 10‐year survival rate of patients with metastatic melanoma [123]. It is widely believed that cancer cells induce CTL apoptosis by expressing PD‐L1. However, recent studies have revealed that CAFs can also express PD‐L1, thereby contributing to CTL apoptosis.

In recent years, the discovery of PD‐L1(+) CAFs in various solid tumors has deepened our understanding of the functions of CAFs. In addition to their previously known role in secreting cytokines to regulate PD‐L1 expression in cancer cells, the direct inhibitory effect of CAFs on CTLs must also be acknowledged. In esophageal cancer, cancer cells and fibroblasts mutually enhance PD‐L1 expression, contributing to tumor immune suppression. In vivo experiments have demonstrated that anti‐PD‐L1 antibodies promote the death of both CAFs and cancer cells, resulting in an increase in CD8+ T cells and a reduction in FoxP3+ Treg cells [124]. Due to cancer heterogeneity, PD‐L1 expression in CAFs is not universal. Teramoto et al. [125] isolated PD‐L1(+) CAFs in NSCLC, observing this subpopulation in 24.8% of patients. Patients with PD‐L1(+) CAFs had a significantly better prognosis compared with those with PD‐L1(−) CAFs. In vitro experiments further demonstrated that PD‐L1 expression in CAFs is reversibly regulated by environmental stimuli, including IFN‐γ produced by activated lymphocytes [125]. Zhang et al. [126] found that Jagged1 derived from glioma enhanced CAF proliferation and increased PD‐L1 expression in vitro. In gliomas with Jagged1 expression, the levels of Notch1, c‐Myc, and PD‐L1 were significantly elevated. Furthermore, it was confirmed that Notch1 and PD‐L1 expression were localized to CAFs in glioma tissues [126]. Although most other studies primarily attribute PD‐L1 expression to cancer cells, we cannot overlook the direct expression of PD‐L1 in CAFs, which may explain the differences in the efficacy of immunotherapy in some cancers. High‐throughput omics technologies, such as single‐cell sequencing, provide an intuitive perspective to observe the role of PD‐L1(+) CAFs in the TME [127, 128].

#### 3.1.2 Recruit Immunosuppressive Cells

Immunosuppressive cells within tumors include various cell types. Extensive research across different tumor types has identified Treg cells, MDSCs, tumor‐associated macrophages (TAMs), and tumor‐associated neutrophils (TANs) as the primary contributors to immune suppression in the TME [104, 106, 129, 130, 131]. These cells foster the immunosuppressive microenvironment by secreting inhibitory cytokines, promoting angiogenesis, and recruiting additional immunosuppressive cells [132, 133, 134]. CAFs can recruit various immunosuppressive cells through the secretion of multiple cytokines.

A major advance in the understanding of immune regulation is the identification and characterization of a group of CD4(+) T cells, known as Treg cells, which play a critical role in preventing autoimmune responses, including organ‐specific autoimmunity, systemic autoimmunity, and colitis. A large number of Treg cells are enriched in tumors, and these cells are highly activated and express elevated levels of coinhibitory molecules such as CTLA‐4, PD‐1, LAG3, and TIGIT [135, 136, 137, 138]. Simultaneously, Treg cells can secrete IL‐10, promoting CTL exhaustion [139]. The specific roles and mechanisms of Treg cells in the tumor immunosuppressive microenvironment have been extensively reviewed. Here, we focus on the impact of CAFs on this T cell population [140]. Research by Costa et al. [21] demonstrated that CAFs attract CD4+ CD25+ T lymphocytes by secreting CXCL12 and CAF‐S1 and retain them through OX40L, PD‐L2, and JAM2. Meanwhile, CAFs can enhance the inhibitory effect of Treg cells on the proliferation of effector T cells. Notably, this work identified four distinct CAF subpopulations in breast cancer, indicating the high heterogeneity of CAFs [21]. apCAFs are a specialized subtype of CAFs derived from mesothelial cells. In pancreatic cancer, mesothelial cells downregulate their mesothelial characteristics and acquire fibroblast‐like features, thereby forming apCAFs. These apCAFs express MHC‐II molecules and can directly interact with naive CD4+ T cells, inducing their differentiation into Treg cells in an antigen‐specific manner [19]. Varveri et al. [141] reported that α‐SMA+ CAFs can form immune synapses with Foxp3+ Treg cells within tumors. These α‐SMA+ CAFs are capable of phagocytosing and processing tumor antigens, exhibiting a tolerogenic phenotype that promotes the antigen‐specific arrest, activation, and proliferation of Treg cells. This study highlighted the immunosuppressive mechanism underlying the synapse formation between α‐SMA+ CAFs and Treg cells, which occurs through an autophagy‐dependent process [141]. Research by O'Connor et al. [142] indicates that CAFs provide TGF‐β during T cell activation, leading to an expansion of activated Treg cells and a subsequent reduction in available IL‐2. Inhibition of TGF‐β signaling can prevent CAF‐driven upregulation of CXCL13 and inhibit the expansion of the Treg cell population [142]. The same phenomenon was observed in SCLC of neuroendocrine origin [143].

However, some studies have shown that specific subgroups of CAFs do not promote Treg cells; instead, they can inhibit Treg cell activity. Zhao et al. [144] identified a novel CAF subgroup in OSCC that is CD68‐positive and highly expresses CD68. The presence of high CD68+ CAFs was linked to tumor initiation. Interestingly, a higher proportion of tumor‐supportive Treg cells was observed in patients with low CD68+ CAFs. Mechanistically, knockdown of CD68 in CAFs led to the upregulation of chemokines CCL17 and CCL22 in tumor cells, thereby enhancing Treg cell recruitment [144]. Similarly, Pei et al. [145] found that CAFs express CD1d and activate NKT cells under stress conditions. These NKT cells are considered crucial players in the body's antitumor immune response [145, 146]. This research highlights the critical role of CAFs in the proliferation and activation of Treg cells within the TME. However, due to the high heterogeneity of CAFs, some studies have also explored their potential in promoting antitumor immunity. Further classification of CAF subgroups and targeting specific markers for treatment may offer a promising approach to enhancing therapeutic efficacy.

MDSCs are an immature myeloid cell population that arises under pathological conditions and is characterized by their ability to suppress T‐cell activity in cancer [147]. CAFs actively recruit MDSCs by secreting cytokines and chemokines, thereby contributing to the establishment of an immunosuppressive TME. For instance, Zhu et al. [148] demonstrated in HCC that CD36(+) CAFs secrete macrophage migration inhibitory factor to promote MDSC accumulation. Additionally, these CAFs enhance PD‐L1 expression through STAT3 signaling, thereby suppressing CD8+ T cell activity and reinforcing the CAF–MDSC‐driven immunosuppressive axis [148]. Similarly, Kumar et al. [149] found that CAFs produce GM‐CSF and IL‐6 to recruit polymorphonuclear MDSCs, which contribute to resistance against CSF1R blockade. This highlights CAF‐driven MDSC regulation as a key mechanism underlying therapy resistance [149]. The crosstalk between CAFs and MDSCs involves multiple signaling pathways. Yang et al. [150] identified STAT3 activation in CAFs as a key driver of CCL2 upregulation, which recruits MDSCs through the CCL2–CCR2 axis and enhances their immunosuppressive functions. In lung squamous cell carcinoma, Xiang et al. [151] demonstrated that CAFs promote the differentiation of monocytic MDSCs through ROS/p38 MAPK pathway activation, establishing a link between oxidative stress and CAF‐mediated immunosuppression. In pancreatic cancer, Bianchi et al. [152] confirmed that CAFs activate the CXCL1/CXCR2 axis to induce neutrophil‐derived TNFα production, promoting an MDSC‐enriched TME and highlighting the pivotal role of chemokine networks in tumor immunosuppression. CAFs also regulate MDSCs indirectly through epigenetic and exosomal mechanisms. Zhao et al. [153] reported in esophageal squamous cell carcinoma that CAF‐derived IL‐6, in synergy with exosomal miR‐21, activates STAT3 signaling, driving MDSC expansion and conferring resistance to cisplatin. Additionally, CAF‐secreted EVs deliver immunosuppressive cargo that directly enhances MDSC‐mediated T‐cell suppression, emphasizing the multifaceted complexity of CAF–MDSC interactions [154].

Targeting the CAF–MDSC axis may enhance immunotherapy efficacy. Khalaf et al. [155] suggested that neutralizing CAF‐derived CCL2 or CXCL12 could help remodel the TME and alleviate immunosuppression. Recent work by Akiyama et al. [156] demonstrated that dual PDGFRα/β blockade reduces MDSC infiltration and synergizes with anti‐PD‐1 therapy, providing a preclinical rationale for combination strategies in cancer treatment. In summary, CAFs recruit and activate MDSCs through secreted factors, signaling pathways, and exosomal communication, forming a cascading immunosuppressive network. Therapeutic cotargeting of this axis has the potential to overcome current limitations in cancer immunotherapy.

TAMs are macrophages that play a crucial role in the formation of the TME and are commonly found in various tumors [157, 158]. TAMs are generally classified into two main subtypes within the TME: M1 and M2. M1 macrophages are involved in antitumor immunity, whereas M2 macrophages promote immunosuppression and support tumor progression [159]. The primary CAF subtypes (iCAF, myCAF, and apCAF) are essential components of the TME. Their interaction with TAMs is primarily mediated through cytokine secretion. iCAFs predominantly contribute to macrophage enrichment, whereas myCAFs play a major role in TAM polarization.

CAFs can recruit monocytes to the tumor site through various signaling pathways. Similar to the recruitment of MDSCs, CAFs may recruit monocytes to the tumor site via the CCL2–CCR2 signaling pathway. In a study on esophageal squamous cell carcinoma, Higashino et al. [160] found that CAFs induce M2 polarization of TAMs, with FAP expression being a key factor driving the tumor‐promoting and immunosuppressive phenotype of CAFs. Similar results were observed in studies on intrahepatic cholangiocarcinoma [150]. The CXCL16 chemokine derived from CAFs may recruit monocytes, promote stromal activation, and facilitate tumor progression in TNBC [161]. Likewise, CXCL14 produced by CAFs can enhance macrophage recruitment to the tumor site, promoting prostate tumor growth [162]. Among the pathways involved in monocyte recruitment, the CXCL12–CXCR4 axis has been extensively studied. CAFs produce high levels of CXCL12 in the TME, and the CXCL12–CXCR4 axis plays a crucial role in recruiting monocytes to the tumor tissues [163]. The iCAF–TAM axis primarily activates the complement cascade pathway through the interaction of complement C5 and its receptor C5AR1. The C5 pathway serves as a crucial chemokine for recruiting immunosuppressive myeloid cells, ultimately leading to the inhibition of T‐cell activity [164]. The interaction of the C3–C3aR iCAF–TAM axis has been further elucidated in melanoma, head and neck cancers, and breast cancer [165]. CD34+ CAFs produce C3 and convert it to its activated form (C3a) in the TME, facilitating the recruitment of C3aR+ circulating monocytes. CAFs primarily promote the M2 polarization of TAMs by secreting cytokines. In summary, a variety of cytokines, including IL‐8, IL‐33, IL‐10, TGF‐β, and CCL2, can be secreted by CAFs, and they have been shown to promote M2 polarization of TAMs in the TME [39, 166, 167, 168, 169].

The role of TANs in cancer progression and tumor immunity remains controversial. In many solid tumors, a high neutrophil‐to‐lymphocyte ratio is associated with poor overall survival (OS). Extensive research indicates that neutrophils suppress both innate and adaptive immunity, thereby facilitating tumorigenesis [170, 171, 172, 173, 174].

Multiple studies have demonstrated that CAFs recruit immunosuppressive neutrophils through specific chemokine signaling pathways, thereby shaping an immunosuppressive TME. In HCC, CAFs were shown to secrete CXCL12, which directly recruits CXCR4+ neutrophils that impair CD8+ T cell function via arginase‐1 (ARG1) production and reactive oxygen species (ROS)‐mediated suppression [6]. Spatial transcriptomic analysis in CRC further revealed neutrophils densely clustered within CAF‐enriched immune hubs, where their spatial colocalization with CAFs correlates with upregulated PD‐L1 expression and adaptive immune resistance [175]. The functional interplay between CAFs and neutrophils extends beyond chemotaxis; in breast cancer models, fibroblast‐derived IL‐33 activates neutrophil ST2 receptors, driving IL‐10/TGF‐β production that polarizes Th2‐type immunity [167]. scRNA‐seq studies in breast cancer additionally identified bidirectional CXCL8–CXCR1/2 axis communication between CAFs and neutrophils, amplifying N2 polarization characterized by ARG1+ and CCL2+ phenotypes that inhibit dendritic cell (DC) maturation [176]. Recent spatial mapping in lung cancer uncovered CAF‐organized fibrotic barriers that spatially confine immunosuppressive neutrophils at tumor‐invasive fronts while excluding cytotoxic T cells, a spatial configuration associated with poor clinical outcomes [177]. Similarly, pancreatic cancer liver metastasis models demonstrated stable ICAM‐1/β2‐integrin‐mediated adhesions between CAFs and neutrophils that cooperatively block T cell transmigration [178]. Clinically, CAF‐mediated neutrophil recruitment mechanisms are particularly significant in premetastatic niche formation, with CRC studies identifying CXCL1/2–CXCR2 and CSF3R as potential therapeutic targets to disrupt this axis [179]. Emerging spatial intervention strategies suggest that disrupting CAF‐neutrophil cluster colocalization could enhance PD‐1 blockade efficacy, evidenced by a 3.2‐fold increase in treatment response in preclinical models through microenvironmental reprogramming [175]. Collectively, these findings position CAF‐neutrophil crosstalk as a critical orchestrator of immunosuppression across multiple malignancies, offering novel therapeutic opportunities through spatial and functional modulation of tumor‐stromal interactions.

In summary, CAFs contribute to the formation of an immunosuppressive TME by recruiting immunosuppressive cells through cytokine secretion and other mechanisms, thereby inhibiting antitumor immunity. However, the diverse functional roles of different CAF subpopulations highlight the complexity of their interactions. Further investigation using omics technologies to elucidate the crosstalk between specific CAF subtypes and immunosuppressive cells is crucial. These insights may pave the way for novel therapeutic strategies targeting CAF‐mediated immunosuppression in cancer immunotherapy.

#### 3.1.3 Secretion Inhibitory Factor

In addition to recruiting immunosuppressive cells through cytokine secretion, CAFs can directly suppress the activity of antitumor immune cells, particularly CD8+ T cells, by releasing immunosuppressive cytokines. TGF‐β, a key cytokine secreted by CAFs, plays a crucial role in shaping the immunosuppressive TME [115, 180]. The activation of the TGF‐β signaling pathway in the TME has been extensively documented in previous reviews [181, 182]. The study by Li et al. [183] demonstrates that Ln‐γ2 is transcribed and activated via the JNK/AP1 signaling pathway in response to TGF‐β1 secreted by CAFs. This process alters T cell receptor expression, thereby preventing T cell infiltration into tumor nests [183]. More importantly, TGF‐β directly suppresses the function of effector T cells, further contributing to immune evasion in the TME. In melanoma, research by Ahmadzadeh et al. [184] demonstrated that TGF‐β1 not only inhibits the acquisition of effector functions in human memory CD8+ T cells and tumor‐infiltrating lymphocytes but also suppresses their expression.

Compared with effector T cells, the antitumor effects of NK cells have been studied to a lesser extent. NK cells can rapidly kill multiple adjacent cells that display surface markers associated with cancer. This unique ability, combined with their capacity to enhance antibody and T‐cell responses, positions NK cells as key anticancer agents [185, 186, 187, 188]. Despite the various mechanisms tumors may develop to evade NK cell attacks, recent advancements in CAR‐NK cell therapy have shown promising potential as a novel treatment approach [189]. CAFs suppress NK cell activation by secreting prostaglandin E2 (PGE2) and indoleamine 2,3‐dioxygenase (IDO), thereby inhibiting key activating receptors such as DNAM‐1, NKp44, and NKp30. This immunosuppressive mechanism has been observed in liver, breast, and CRCs [190, 191, 192]. Studies have demonstrated that CAF inhibitors can enhance the efficacy of CAR‐NK cell therapy by reducing IL‐6 secretion from CAFs, thereby mitigating immunosuppressive effects in the TME [193]. A study has also shown that in gastric cancer, CAFs induce ferroptosis in NK cells by regulating iron metabolism, which contributes to immune evasion [194]. In breast cancer, a distinct subpopulation of senescent CAFs secretes a specialized ECM that specifically impairs NK cell cytotoxicity, thereby facilitating tumor progression [64]. Similarly, CAFs express Dickkopf‐1, a Wnt/β‐catenin inhibitor, which suppresses NK cell activation and cytotoxicity by downregulating AKT/ERK/S6 phosphorylation [195]. Remarkably, CAFs can induce their own lysis and downregulate the expression of activating receptors on NK cells through ligand–receptor interactions. This process promotes cancer cell evasion from NK cell surveillance, further contributing to tumor immune escape [196].

In PDAC, NetG1‐associated CAFs demonstrate intrinsic immunosuppressive properties that inhibit NK cell‐mediated tumor cell killing. This suppression is regulated by the NetG1 downstream signaling pathway, which involves AKT/4E‐BP1, p38/FRA1, vesicular glutamate transporter 1, and glutamine synthetase [197]. Similar inhibitory effects have also been observed in endometrial cancer [198]. Notably, radiotherapy does not eliminate this suppression, as CAFs maintain their ability to inhibit NK cell activity even after treatment [199].

Recent studies have demonstrated that CAFs inhibit NK cells through various mechanisms. This complexity is evident not only in the heterogeneity across different tumor types but also in the diverse regulatory pathways within the same tumor, as seen in breast cancer. Such variability poses a challenge to the precision of tumor therapy. A potential strategy to enhance treatment efficacy is to consider multiple pathways when selecting therapeutic targets, thereby overcoming CAF‐mediated immune suppression.

**FIGURE 2:**
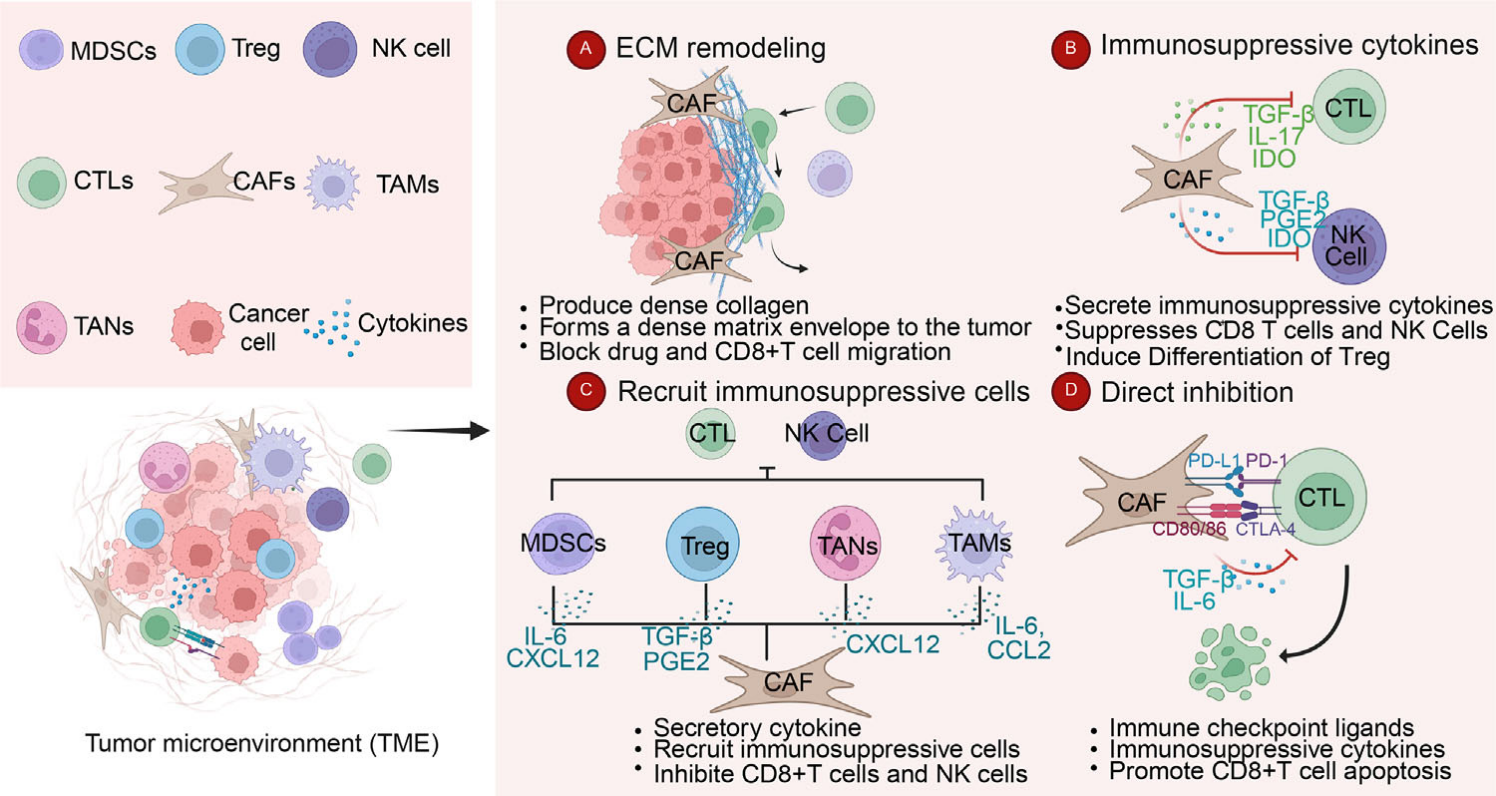
Immunosuppressive mechanisms of cancer‐associated fibroblasts (CAFs) in the tumor microenvironment (TME). This schematic illustrates the various ways in which CAFs contribute to immune evasion within the TME. (A) Extracellular matrix (ECM) remodeling: CAFs produce dense collagen and establish a matrix barrier around the tumor, hindering the infiltration of CD8⁺ T cells and therapeutic agents. (B) Immunosuppressive cytokines: CAFs secrete cytokines such as TGF‐β, IL‐17, and IDO, which suppress CD8⁺ T cells and NK cells while promoting the differentiation of regulatory T cells (Tregs). (C) Recruitment of immunosuppressive cells: CAFs release cytokines (e.g., TGF‐β, PGE2, IL‐6, and CXCL12) that attract myeloid‐derived suppressor cells (MDSCs), Tregs, tumor‐associated neutrophils (TANs), and tumor‐associated macrophages (TAMs), further suppressing antitumor immunity. (D) Direct inhibition: CAFs express immune checkpoint ligands such as PD‐L1 and CTLA‐4, which interact with CD8⁺ T cells to induce apoptosis and impair their cytotoxic function. This figure emphasizes the multifaceted immunosuppressive role of CAFs in tumor progression and resistance to immunotherapy (created in https://BioRender.com).

Regulating the immune microenvironment of tumors is a critical function of CAFs. By secreting cytokines to recruit immunosuppressive cells, directly inhibiting antitumor immune cells, and expressing immune checkpoints, CAFs play a pivotal role in maintaining the immunosuppressive TME. However, the mechanisms involved are highly complex, with significant heterogeneity observed not only between different tumor types but also among subtypes within the same tumor. To counter the immunosuppressive effects of CAFs, multiple immunotherapeutic approaches should be employed to target as many pathways as possible while also developing precise therapies aimed at specific CAF subtypes. Simultaneously, the potential dual roles of CAFs in both promoting and inhibiting tumors must be carefully considered, and further research is needed to better understand the underlying mechanisms (Figure 2).

### 3.2 Matrix Remodeling and Angiogenesis

In various tumors, including breast [79, 200], head and neck [201, 202], esophageal [203], pancreatic [204], and CRCs [205], a dense, rigid ECM with highly cross‐linked fibers is associated with increased metastatic potential and poor prognosis [206, 207, 208]. The dense ECM obstructs drug delivery by acting as a physical barrier to compound penetration [209, 210] and by compressing blood vessels. This vascular compression reduces the supply of oxygen and nutrients, ultimately impairing lymphocyte activation [211, 212]. Angiogenesis, the formation of new blood vessels from preexisting vasculature, is essential for tumor growth and hematogenous metastasis. CAFs play a critical role in this process by secreting proangiogenic growth factors, attracting vascular endothelial cells, and recruiting monocytes from the bone marrow. Additionally, the ECM produced by CAFs modulates the biomechanical properties of the tumor stroma, including stiffness, elasticity, and interstitial fluid pressure. These changes indirectly influence tumor vascularization and blood flow, further supporting tumor progression. As a result, therapeutic approaches targeting CAFs or indirectly targeting the ECM and tumor vessels generated by CAFs have garnered significant attention in recent years.

#### 3.2.1 Matrix Production and Remodeling

Activation of the TGF‐β pathway induces the transformation of fibroblasts into CAFs within the TME [115, 213]. CAFs involved in ECM deposition exhibit myofibroblast‐like characteristics and express ECM‐related genes, including those encoding collagen, proteoglycans, and glycoproteins [214, 215]. Additionally, CAFs secrete enzymes such as LOXs and hydroxylases, which catalyze the cross‐linking of collagen and elastin, as well as the posttranslational modifications of ECM proteins, thereby contributing to the structural and mechanical properties of the tumor stroma [83, 216].

The deposited ECM contributes to tumor drug resistance by acting as a physical barrier, limiting drug penetration and reducing therapeutic efficacy. In breast cancer, high‐density stroma significantly suppresses T‐cell proliferation compared with low‐density stroma. Prolonged culture in a high‐density stromal environment leads to an increased CD4+/CD8+ T‐cell ratio. Notably, the surrounding collagen density does not influence cancer cell proliferation [217]. Under the mechanical pressure exerted by a rigid ECM, infiltrating T lymphocyte populations undergo significant alterations in quantity, surface marker expression, subset composition, and gene expression profiles [218]. The motility of cancer cells is inherently limited, preventing them from traversing the ECM solely through their own mechanisms [219]. Studies have shown that CAFs expressing α‐SMA can acquire the ability to contract the ECM within the TME. This contractile capability facilitates tumor cell penetration through the dense ECM, thereby promoting tumor invasion [220, 221]. This clarifies why the dense ECM exerts a greater impact on immune cells than on tumor cells and how CAFs facilitate tumor cell migration within the ECM.

The rigidity of the ECM also influences various immune cells, modulating their infiltration, activation, and functional properties within the TME. Studies have shown that the dense ECM in tumors not only directly suppresses the activity of CD8+ T cells but also affects the function of immunosuppressive cells, such as TAMs, thereby enhancing their inhibitory effects on T cells [222, 223]. The primary component of the ECM is collagen. In an in vivo model, knocking out the collagen gene resulted in a decreased OS rate [224, 225]. However, when a LOX inhibitor was combined with the chemotherapy drug gemcitabine for the treatment of early‐stage PDAC, it improved OS in mice by reducing metastasis [226]. These findings suggest that ECM remodeling may have a dual role in promoting tumor invasion while also restricting tumor progression.

The ECM facilitates paracrine signaling with tumor cells, activating a series of key biochemical pathways associated with tumor invasion and aggressiveness. These activated pathways include FAK, WNT, and MAPK, leading to EMT, disrupting cell polarity, and ultimately resulting in increased tumor proliferation and invasive capabilities. In the paracrine mechanism, EVs serve as crucial mediators. EVs secreted by CAFs promote tumor invasion and metastasis through multiple pathways, including exosomes and secreted proteins, by activating the FAK pathway [83, 227, 228]. Similarly, the activation of the FAK pathway, the WNT pathway plays a significant role in tumor invasion and metastasis. Through the mediation of the ECM, the activated WNT pathway can enhance tumor invasion by strengthening the stemness of cancer cells and promoting EMT [229, 230, 231]. The MAPK pathway is not only a key signaling pathway activated by the paracrine mechanisms of CAFs, but studies have also shown that its activity can distinguish different CAF subtypes in PDAC, highlighting its crucial role in paracrine signaling. Furthermore, MAPK pathway activation is associated with poor prognosis in ovarian cancer and promotes invasion and metastasis in lung cancer [232, 233, 234, 235].

CAFs promote tumor invasion and metastasis by secreting ECM components and through ECM‐mediated paracrine signaling. However, under certain conditions, a stiff ECM can paradoxically restrict tumor cell invasion, an intriguing phenomenon observed in numerous studies. This highlights the challenges associated with simply eliminating ECM‐producing CAFs or employing ECM‐targeted therapies. The contractile effect of CAFs within the TME warrants particular attention, as it represents a mechanism by which tumor cells regulate ECM stiffness. Disrupting this contractile effect could potentially leverage the ECM to suppress tumor cell invasiveness, thereby transforming CAFs into therapeutic allies. Additionally, studies indicate that reducing the primary ECM component, collagen, does not effectively treat tumors. Instead, targeting enzymes responsible for collagen cross‐linking may offer a more promising approach. Similarly, inhibiting the complex paracrine signaling pathways within the ECM could theoretically mitigate fibroblast‐induced tumor progression, but identifying precise therapeutic targets is essential. In this regard, spatial omics provides a powerful tool for gaining deeper insights into ECM dynamics and fibroblast–tumor interactions.

#### 3.2.2 Angiogenesis

CAFs play a crucial role in tumor angiogenesis. In tumors, CAFs and tumor‐associated blood vessels (TABVs) have been observed to colocalize [236, 237]. CAFs serve as the primary source of tumor‐derived VEGFA; however, they can also support tumor angiogenesis through VEGFA‐independent mechanisms [238, 239, 240, 241]. For instance, CAF‐derived PDGFC sustains angiogenesis by stimulating the secretion of proangiogenic growth factors such as FGF2 and osteopontin [242, 243, 244]. Furthermore, the CAF secretome enhances tumor angiogenesis by attracting vascular endothelial cells and recruiting monocytes from the bone marrow. Studies have also demonstrated that CAFs promote angiogenesis by activating the PI3K/AKT and TGF‐β signaling pathways, further underscoring their critical role in tumor vascularization [92, 245]. The integrated stress response (ISR) is a homeostatic mechanism that links cell growth and survival to bioenergetic demands. Verginadis et al. [95] demonstrated that the activation of the ISR stimulates CAFs surrounding blood vessels, thereby driving tumor angiogenesis. Tumor‐associated stromal cells, derived from mesenchymal tissues, play a crucial role in the formation of TABVs, with CAFs serving as key mediators. CAFs contribute to the TME by providing the ECM, secreting cytokines, and recruiting bone marrow‐derived cells. Currently, antiangiogenic therapies primarily target VEGF and VEGFR, and in certain cancer types, their combination with chemotherapy or immunotherapy has significantly improved survival outcomes [246]. Enhancing treatment efficacy by integrating more precise therapeutic strategies targeting CAFs holds great promise and warrants further investigation.

### 3.3 CAFs in Tumor Metastasis

Malignant tumor metastasis is a complex process in which tumor cells evade immune surveillance, infiltrate the bloodstream or lymphatic system from the primary site, and establish colonies in specific organ tissues [247, 248]. Distant metastasis is strongly associated with poor prognosis, and in most solid tumors, its occurrence indicates the loss of surgical treatment opportunities [249, 250]. The role of CAFs in tumor proliferation has been previously reviewed. This section specifically examines the pathogenic mechanisms through which CAFs contribute to tumor cell invasion into blood vessels and the formation of the premetastatic niche (PMN).

The penetration of tumor cells through the basement membrane barrier between epithelial and endothelial cells, followed by their entry into blood vessels, adhesion to endothelial cells, and subsequent invasion of distant tissues through the endothelium, represents a critical step in tumor metastasis [251, 252]. CXCL12 plays a crucial role in the intravasation of tumor cells into blood vessels. Secreted by CAFs, it enhances tumor cell intravasation through a synergistic interaction with macrophages [253, 254]. Additionally, CXCL12 promotes tumor angiogenesis and recruits new vascular endothelial cells, thereby increasing vascular permeability and further facilitating tumor cell entry into the bloodstream [255, 256, 257, 258]. PDGF also promotes tumor cell infiltration by activating CAFs to secrete VEGFA and VEGFC. This process enhances lymphangiogenesis and boosts tumor cell infiltration [259, 260, 261, 262].

The extravasation of tumor cells from blood vessels is regarded as the rate‐limiting step in tumor metastasis. MicroRNAs (miRNAs) in EVs are considered key facilitators of tumor cell extravasation. Studies have shown that the activation of the IL‐6/STAT3 signaling pathway in tumor stromal cells, including CAFs, induces the secretion of miRNA‐214 in stromal‐derived EVs, thereby promoting tumor cell extravasation [263]. Additionally, EVs can construct the PMN in situ [264]. Meanwhile, FN1 secreted by CAFs enhances the expression of intercellular adhesion molecule 1, increases endothelial cell permeability, and facilitates the transendothelial migration of tumor cells, further promoting tumor cell extravasation [265, 266]. This complex transfer cascade reaction offers numerous potential therapeutic targets, and better therapeutic effects may be achieved by targeting the infiltration and exudation of tumor cells.

**FIGURE 3:**
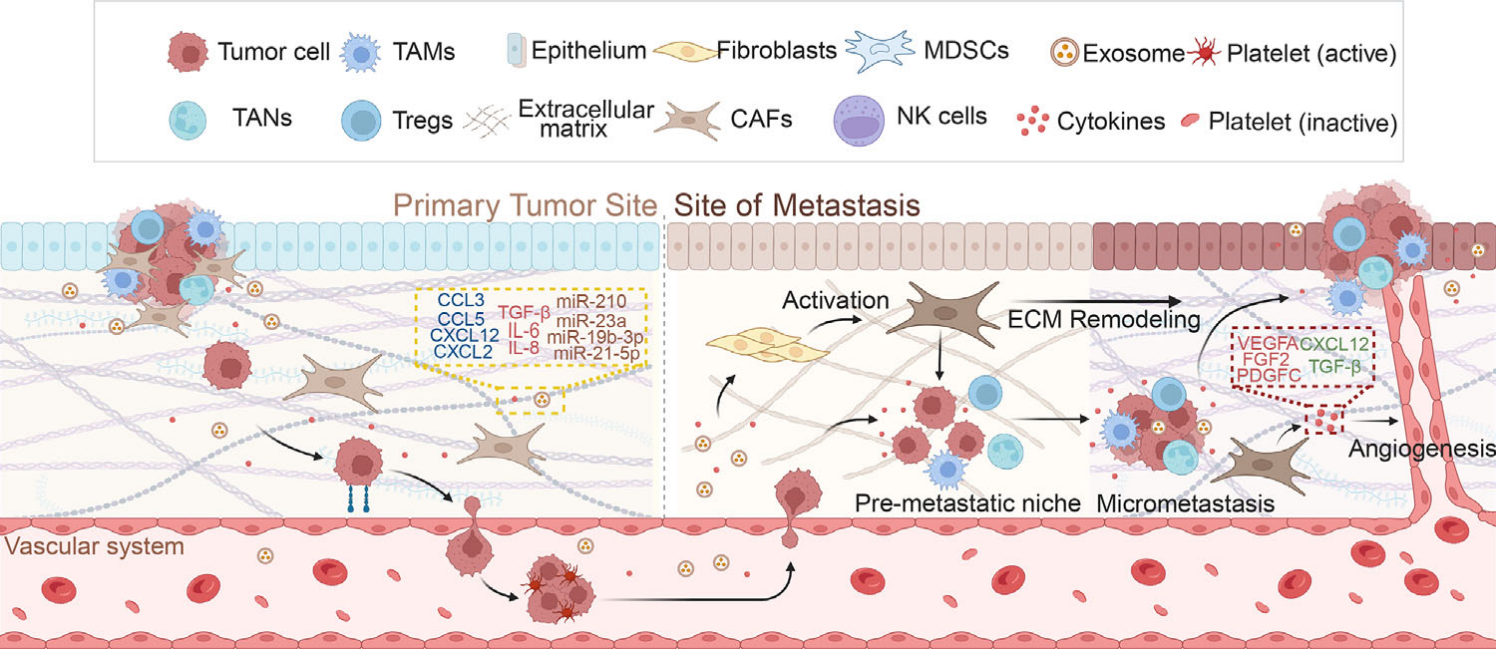
Mechanisms of cancer‐associated fibroblasts (CAFs) in cancer metastasis. The Primary Tumor Site (left panel) highlights key constituents, including tumor cells, tumor‐associated macrophages (TAMs), regulatory T cells (Tregs), myeloid‐derived suppressor cells (MDSCs), fibroblasts, and activated/inactive platelets. Tumor‐derived exosomes and cytokines (e.g., TGF‐β, IL‐6, CCL3/5, and CXCL12) facilitate immune suppression, extracellular matrix (ECM) remodeling, and platelet activation. Activated platelets protect circulating tumor cells during intravasation into the vascular system. The Metastatic Site (right panel) depicts premetastatic niche formation via ECM remodeling, angiogenesis (mediated by VEGFA and PDGFC), and recruitment of immunosuppressive cells. Micrometastases arise through interactions between disseminated tumor cells, cytokines (e.g., IL‐8 and CXCL2), and stromal components like CAFs. MicroRNAs (miR‐210, miR‐21, and miR‐23a) carried by exosomes further reprogram the metastatic microenvironment to support tumor cell survival and proliferation (created in https://BioRender.com).

Under normal conditions, the ECM lacks the nutrients and cytokines essential for tumor cell proliferation, while immune surveillance remains active. Consequently, tumor cells that extravasate from blood vessels encounter significant challenges in establishing colonization within the matrix. Studies have shown that EVs and cytokines released by tumor cells can travel through the bloodstream to metastatic sites and contribute to PMN formation. This occurs through multiple mechanisms, including enhancing vascular permeability, altering mesenchymal cell properties, and remodeling the ECM [267]. EVs transport RNA and proteins that induce the transformation of tissue‐resident mesenchymal cells into metastasis‐associated fibroblasts (MAFs) [268, 269, 270]. Similar to CAFs, the activation of MAFs is closely related to the TGF‐β, PDGFRα, and IL‐1α/β pathways, ultimately leading to various pathogenic mechanisms mentioned earlier, such as immunosuppression and stromal remodeling [271, 272, 273]. Although MAFs originate from different tissues than CAFs at the primary tumor site, they exhibit similar functions. Moreover, highly heterogeneous MAF populations have been identified across multiple metastatic sites. MyoMAFs, inflammatory MAFs, and mesothelial MAFs have been identified in liver metastases of colorectal and pancreatic cancers [10]. Research on MAFs in metastatic foci remains limited due to challenges such as sample accessibility, particularly in studies utilizing omics approaches to analyze the immune microenvironment. This limitation constrains our understanding of MAFs and their role in PMN formation. Nevertheless, like CAFs, MAFs are critical prometastatic cells that warrant further investigation (Figure 3).

## 4 CAF as a Diagnostic Biomarker: An Imaging Target

CAFs are a crucial component of the TME and play a significant role in tumor invasion, metastasis, and drug resistance. As a biomarker for diagnosis, CAFs have garnered considerable research attention, primarily focusing on the development of imaging techniques that target specific biomarkers of CAFs and the imaging of CAF‐related ECM. FAP, as a specific biomarker of CAFs, is among the most promising tumor imaging markers, including PET, fluorescence, and multimodal molecular imaging targeting FAP. Current clinical trials imaging CAFs are summarized in Table 1.

**TABLE 1:** Clinical application of CAFs in cancer diagnosis and prognosis.

Clinical trial number(s)	Research title	Research stage	Target	Probe	Diagnosis/prognosis	Cancer type(s)	Outcomes
NCT04621435, NCT05180162	Imaging of solid tumors using FAP‐2286	I	FAP	^68^Ga/^64^Cu–FAP‐2286	Diagnosis	Metastatic cancer (solid tumors)	The detection capability of [^68^Ga]Ga–FAP‐2286 PET for primary lesions, distant metastases, and lymph node metastases is superior to that of ^18^F‐FDG.
NCT05543317	^68^Ga‐FAPI‐RGD PET/​CT for dual integrin αvβ3 and FAP‐targeted imaging in patients with various types of cancer and compared with ^18^F‐FDG	Not applicable	FAP	^68^Ga–FAPI–RGD	Diagnosis	Solid tumors	^68^Ga–FAPI–RGD PET/CT facilitates visualization of tumor lesions with favorable imaging contrast.
NCT04147494	Experimental PET imaging scans before cancer surgery to study the amount of PET tracer accumulated in normal and cancer tissues	I	FAP	^68^Ga–FAPI–46	Diagnosis	Various cancer types	The SUV and immunohistochemical score of [^68^Ga]FAPI‐46 are higher in cancerous tissues than in adjacent noncancerous tissues. The FAP immunohistochemical score is closely related to the average SUVmax and SUV values of [^68^Ga]FAPI‐46.
NCT05365802	PET study of ^68^Ga‐FAPI‐46 in Patients with interstitial lung disease: an exploratory biodistribution study with histopathology validation	I	FAP	^68^Ga–FAPI‐46	Diagnosis	Interstitial lung disease	FAPI uptake was primarily visualized in the fibrotic area on CT.
NCT04554719, NCT04605939	Clinical application of fibroblast activation protein PET/MRI for diagnosis and staging	Not applicable	FAP	^68^Ga–FAPI–04	Diagnosis	Malignant tumors	When combined with MRI, [^68^Ga]FAPI‐04 PET/MR has the potential to reduce the misdiagnosis of certain pancreatic lesions.
NCT05430841	Evaluating the potential usefulness of ^18^F‐AlF‐FAPI PET/CT in patients with gastrointestinal tumors and compared with ^18^F‐FDG PET/CT	Not applicable	FAP	^18^F‐FAPI‐74	Diagnosis	Gastrointestinal cancers	[^18^F]FAPI‐74 PET/CT is superior to [^18^F]FDG PET/CT in detecting primary tumors, local recurrence, lymph node involvement, and bone and visceral metastases in gastric, pancreatic, and liver cancers, exhibiting higher uptake in most primary and metastatic lesions.
NCT04504110	A prospective study evaluating ^68^Ga‐FAPI‐04 and ^18^F‐FDG PET/CT in patients with epithelial ovarian cancer: comparison with histological results	II	FAP	^68^Ga–FAPI‐04	Diagnosis	Epithelial ovarian cancer	Undergoing
NCT04459273	PET biodistribution study of ^68^Ga‐FAPI‐46 in patients with different malignant tumors: an exploratory biodistribution study with histopathological validation	I	FAP	^68^Ga–FAPI‐46	Diagnosis	Various cancer types	Undergoing
NCT05641896	A phase 2, multicenter, single arm, open label, non‐randomized study of [^18^F]FAPI‐74 PET in patients with gastrointestinal cancers	II	FAP	^18^F‐FAPI‐74	Diagnosis	Gastrointestinal cancers	Undergoing
NCT05262855	A phase 2, multicenter, single arm, open label non‐randomized study of [^68^Ga]FAPI‐46 PET in patients with resectable or borderline resectable pancreatic ductal carcinoma	II	FAP	^68^Ga–FAPI‐46	Diagnosis	Resectable pancreatic ductal carcinoma	Undergoing
NCT05784597	A phase I study to evaluate the safety and dosimetry of ^68^Ga‐labelled OncoFAP derivatives in patients with solid tumors	I	FAP	^68^Ga–OncoFAP	Diagnosis	Breast cancer, colorectal cancer, esophageal cancer, and pancreatic adenocarcinoma	Undergoing
NCT03465722	(VOYAGER) Study of avapritinib vs regorafenib in patients with locally advanced unresectable or metastatic GIST	III	PDGFR	KIT/PDGFRA ctDNA	Prognosis	ctDNA sequencing efficiently detects KIT/PDGFRA mutations and prognosticates outcomes in patients with TKI‐resistant gastrointestinal stromal tumor (GIST) treated with avapritinib.	Undergoing

*Data source*: https://clinicaltrials.gov/.

*Abbreviations*: FAP: fibroblast activation protein, PET: positron emission tomography, CT: computed tomography, MRI: magnetic resonance imaging, SUV: standardized uptake value, PDGFR: platelet‐derived growth factor receptor, ctDNA: circulating tumor DNA, TKI: tyrosine kinase inhibitor, FDG: fluorodeoxyglucose, FAPI: fibroblast activation protein inhibitor.

### 4.1 Imaging FAP

FAP is a membrane‐bound type II serine protease that is highly expressed in CAFs. It has a relative molecular mass of 97 kDa and forms a dimer composed of a 95 kDa α‐subunit and a 105 kDa β‐subunit [274]. As a member of the DPP4 family, FAP hydrolyzes peptide hormones and contributes to tumor progression by degrading the ECM, thereby promoting invasion and metastasis [275, 276, 277]. Notably, FAP exhibits minimal expression in normal tissues but is highly abundant in the stroma of over 90% of malignant epithelial tumors, making it a promising target for both pan‐cancer therapy and tumor imaging [278, 279].

#### 4.1.1 Positron Emission Tomography

PET combined with computed tomography (CT) is a widely utilized nuclear medicine technique that uses positron‐emitting radioisotope‐labeled compounds to visualize and evaluate biochemical processes in vivo [280]. This integration offers critical insights into both metabolic activity and the precise anatomical localization of diseases. The most commonly used tracer, fluorine‐18 fluorodeoxyglucose (^18^F‐FDG), facilitates the visualization of glucose uptake in tumors and metastases. Since cancer cells exhibit increased metabolic activity, they absorb more tracer, appearing as brighter regions on imaging [281, 282, 283]. However, ^18^F‐FDG PET has limitations in distinguishing malignant tumors from benign inflammatory or infectious processes due to the nonspecific uptake of glucose [280]. In contrast, FAP inhibitor (FAPI) tracers function independently of glucose metabolism, minimizing background signals in regions such as the stomach, intestines, and lungs [284]. FAPI exhibits stable uptake, high image quality, and superior clinical performance across various tumor types, positioning it as a promising alternative to ^18^F‐FDG for oncologic imaging. Various enzyme inhibitors targeting FAP, including the widely used FAPI‐04 and FAPI‐46, as well as the more selective UAMC‐1110, have been thoroughly discussed in previous reviews [284]. In this section, we highlight recent advances in the field, focusing on the novel small‐molecule tracer OncoFAP, which represents a promising development in FAP‐targeted imaging and therapy.

The use of peptide and pseudo‐peptide‐based targeting methods for FAP represents a promising alternative to enzyme inhibitors and has the potential for clinical application. OncoFAP is a highly specific ligand for FAP, offering broad tumor‐targeting capabilities. In animal models, Millul et al. [285] reported that OncoFAP demonstrated effective targeting of tumors. Backhaus et al. [286] conducted an experiment involving 12 patients with various types of tumors to evaluate the use of ^68^Ga–OncoFAP–DOTAGA–PET/CT and PET/MRI, which showed favorable biodistribution and kinetics. The tracer demonstrated high and reliable uptake rates in primary cancers, binding to human FAP at sub‐nanomolar concentrations; due to its low molecular weight, the tracer accumulated rapidly and displayed low immunogenicity [286]. It is noteworthy that relatively successful preclinical studies have been reported for applications in both PET/CT and targeted radionuclide therapy (TRT). In a preclinical study, the modified ^177^Lu–OncoFAP (OncoFAP‐23) showed improved targeting and higher tumor uptake [287]. OncoFAP combined with IL‐2 targeting resulted in increased tumor uptake [288]. While it may be premature to suggest that OncoFAP will replace the established FAPI‐04, its development warrants close attention. Clinical case reports on OncoFAP have demonstrated promising efficacy and safety [289, 290]. However, several key challenges must be addressed before it can be broadly adopted in clinical practice. The first challenge is scalability. While dimeric and trimeric forms of OncoFAP exhibit improved tumor retention, their synthesis involves complex chemical coupling steps that require stringent control over process stability and purity, potentially limiting feasibility for large‐scale production. The second challenge involves regulatory hurdles. Clinical translation of OncoFAP necessitates progression through multiple trial phases to establish safety and efficacy. Phase III trials, in particular, require large patient cohorts and extended follow‐up periods, resulting in considerable time and financial investment. Furthermore, variations in regulatory frameworks and approval standards across countries may prolong the review process. The third challenge is high production costs. The synthesis of OncoFAP relies on high‐purity intermediates, with significant expenses associated with raw materials and purification procedures. Addressing these three challenges will be critical for advancing the clinical development and widespread application of OncoFAP.

The imaging efficacy of FAP‐targeted imaging is closely linked to the abundance of FAP in specific cancers. FAPI demonstrates strong imaging performance in cancers characterized by high stromal content, exhibiting uptake comparable to the commonly used ^18^F‐FDG PET/CT, but with a higher tumor‐to‐background ratio (TBR), particularly in the assessment of brain metastases. However, in cancers with high heterogeneity, such as lung cancer, FAPI has not yet demonstrated a clear advantage in imaging primary lesions. Nonetheless, it offers significant benefits in imaging metastases and distinguishing malignant tumors from benign lesions.

The diagnostic efficacy of FAPI PET/CT in breast cancer has been extensively investigated. Several clinical studies have demonstrated the advantages of FAPI PET/CT over ^18^F‐FDG PET/CT, particularly regarding uptake and TBR. Sahin et al. [291] examined the advantages of ^68^Ga–FAPI PET/CT in imaging invasive lobular breast cancer (ILC). Their retrospective analysis of 23 female ILC patients with hormone‐positive, HER2‐negative tumors showed that ^68^Ga–FAPI PET/CT had higher TBR and SUVmax in primary tumors, as well as improved sensitivity and uptake for detecting axillary lymph nodes and distant metastases [291]. In a separate study involving 20 female breast cancer patients, FAPI imaging demonstrated significantly higher SUVmax and TBR values for primary tumors, lymph nodes, lung, and bone metastases compared with FDG [292]. Elboga et al. [293] compared ^68^Ga–FAPI‐04 and ^18^F‐FDG PET/CT in 48 breast cancer patients and found ^68^Ga–FAPI PET/CT to be superior in detecting primary breast cancer SUVmax. Zheng et al. [283] also confirmed FAPI PET/CT's superiority over FDG in detecting primary breast tumors based on SUVmax and TBR. Although Ballal et al.’s study [294] of 47 breast cancer patients using ^68^Ga–DOTA.SA. FAPI did not show significant differences in some indicators, it still outperformed ^18^F‐FDG overall. The high density of stromal components and the abundant presence of CAFs in breast cancer, along with the stable expression of FAP, are likely key factors contributing to the excellent imaging performance of FAPI PET in this tumor type [97, 295].

In contrast to breast cancer, the situation regarding lung, gastric, and head and neck cancers differs. Due to tumor heterogeneity, FAPI did not demonstrate a significant advantage in the uptake of primary lesions. However, it showed a clear benefit over ^18^F‐FDG PET/CT in terms of the TBR, with this advantage being particularly pronounced in metastatic lesions. FAPI PET/CT and FDG PET/CT exhibited no significant differences in imaging primary lung cancer. In a study of 34 advanced LC patients, Wang et al. [296] found no significant differences in delineating the primary tumor and metabolic volume between the two methods. Similarly, Giesel et al. [297], in a study of 41 primary tumors (including nine LC patients), observed no significant difference in uptake between FAPI and FDG at the primary tumor level (*p* = 0.429), although the comparison did not include uptake rates among LC patients. A prospective analysis by Wu et al. [298] in 28 newly diagnosed NSCLC patients also found no difference between FAPI PET/CT and FDG PET/CT in detecting LC. The high heterogeneity of lung cancer may lead to variable FAP expression among different patients, which could explain the inconsistent imaging results observed with FAPI.

Due to the limited number of human trials available for FAPI PET/CT, both in terms of case volume and large‐scale comparative studies, assessing its effectiveness for specific types of cancer remains challenging. In gastric, head and neck, and CRCs, FAPI has demonstrated superior imaging performance compared with ^18^F‐FDG PET/CT in certain clinical trials. However, due to the limitations in study scale, it is premature to conclude that FAPI can replace ^18^F‐FDG. The imaging performance of FAPI PET/CT in gastric cancer appears to vary across different studies. In a 2022 study by Lin et al., ^68^Ga–DOTA–FAPI‐04 PET/CT showed superior detection efficacy compared with ^18^FDG PET/CT in 56 patients with long‐term proven gastric carcinomas, as evidenced by a higher SUVmax (10.3 vs. 8.1, *p* = 0.004) and TBR (11.6 vs. 5.8, *p* < 0.001) in the primary tumor. Similar results were found in two other studies [299, 300]. However, Kuten et al. [301] conducted a clinical study involving 10 patients with gastric adenocarcinoma undergoing initial staging. They found that, although ^68^Ga–FAPI‐04 PET/CT demonstrated a significant advantage in TBR values compared with ^18^FDG PET/CT (*p* = 0.007), there was no significant difference in SUVmax (*p* = 0.139) [301]. Another study compared the performance of ^68^Ga–FAPI‐04 PET/CT with ^18^F‐FDG PET/CT in 21 patients with pathologically proven newly diagnosed or recurrent gastric adenocarcinoma. For primary gastric cancer, no significant differences were observed in SUVmax (*p* = 0.247) and TBR (*p* = 0.117) between the two groups. Interestingly, FAPI demonstrated significantly better imaging performance for other metastases, including lymph nodes, peritoneum, bone metastasis, and liver metastasis [302]. Contradictory results have also been observed in head and neck cancers. While several studies have demonstrated the effectiveness of FAPI in imaging these cancers [303, 304], other studies suggest that FAPI does not provide a significant advantage over ^18^F‐FDG [305, 306]. In the diagnosis of CRC, the diagnostic value of FAPI PET/CT is evaluated for both the primary lesion and disease staging, focusing on parameters such as SUVmax and TBR [307, 308]. However, some comparative studies have highlighted the nonsignificance of certain key parameters, indicating the need for more clinical research to validate the effectiveness of FAPI in CRC [309, 310, 311]. Studies utilizing FAPI PET/CT have also been reported in the diagnosis of other tumors, such as cholangiocarcinoma [312], cervical cancer [313], HCC [314], pancreatic neuroendocrine tumors [315], and glioma [316, 317]. However, due to the limited number of cases, no definitive conclusion can be drawn regarding the effectiveness of FAPI in these cancers.

Metabolic variability in the liver is likely a key factor influencing the stability of FAPI PET tracers. To address this challenge, optimizing the molecular structure and introducing functional modifications represent promising strategies. Specifically, the incorporation of rigid linkers [318] and metabolic blocking groups may help reduce susceptibility to hepatic enzyme degradation and minimize nonspecific liver uptake. In parallel, the development of multimeric probes and refinement of albumin‐binding strategies can further decrease liver accumulation while enhancing tumor‐specific uptake, thereby improving the overall imaging performance of FAPI tracers [319].

FAPI PET has demonstrated superior performance over FDG PET in detecting metastatic lesions, particularly in the brain, digestive tract, and liver, primarily due to its higher TBR. This advantage is largely attributed to the limitations of FDG PET in regions with high physiological glucose metabolism, which can obscure lesion detection. Additionally, FDG PET is less effective in identifying low‐metabolic tumors, as these lesions exhibit minimal glucose uptake. In contrast, the relatively stable expression of FAP within such lesions enables FAPI PET to offer improved sensitivity and diagnostic accuracy in detecting both high‐ and low‐metabolic metastatic sites [296, 320, 321].

In conclusion, FAPI PET/CT is an emerging and promising diagnostic tool for cancer, demonstrating its diagnostic value in multiple clinical studies across various cancer types. However, current research is insufficient to support its adoption as a guideline‐altering imaging method to replace ^18^F‐FDG PET/CT. The variability in expression across different cancer types may limit its utility as a broad‐spectrum diagnostic approach. Nevertheless, its superior performance in certain cancers suggests the potential to replicate the success of PSMA PET/CT in prostate cancer.

#### 4.1.2 Fluorescence Imaging

In addition to the aforementioned PET/CT imaging, fluorescence visualization technology plays a crucial role in medical research and clinical applications, particularly in tumor imaging and treatment navigation. Fluorescent probes have emerged as indispensable research tools [322] in contemporary chemical biology and biomedical fields due to their advantages, including high spatiotemporal resolution, noninvasiveness, and in situ visualization. These probes have been successfully applied to the dynamic visualization of important biomarkers in living cells and organisms, as well as to the precise diagnosis and treatment of major human diseases, such as in fluorescence‐guided surgery [323].

Early surgical resection of tumors is often the optimal choice for treating diseases. However, there remains a local recurrence rate of 15–20% within the first 5 years after surgery for lung cancer [324, 325]. During surgery, accurately identifying and delineating the tumor margin is crucial for ensuring complete resection and minimizing the risk of recurrence [326]. However, traditional visual inspection and imaging methods have limitations in accurately depicting the tumor boundary, especially for tumors that are challenging to detect or have complex shapes. Intraoperative molecular imaging (IMI) offers a solution by providing real‐time, high‐resolution images of the tumor and its surroundings during surgery [327, 328]. By utilizing tumor‐targeting fluorescent dyes and ultra‐sensitive camera systems, surgeons can visualize the tumor margin with unprecedented clarity. This capability enables more precise surgical resection, reducing the risk of leaving behind microscopic tumor cells and improving patient outcomes.

Early‐generation FAP‐targeted probes, such as FAPI‐04, exhibited limitations for fluorescence imaging due to rapid blood clearance and short tumor retention times. To overcome these challenges, probes modified with albumin‐binding moieties, such as 4‐(p‐iodophenyl)‐butyric acid or Evans blue (EB), were developed to prolong systemic circulation and enhance tumor accumulation [329, 330, 331]. Additionally, incorporating hydrophilic peptide linkers has been employed to reduce off‐target organ retention and improve overall pharmacokinetic profiles. Recent research has shifted toward expanding the utility of these probes beyond imaging alone, exploring their potential in therapeutic applications. For example, fluorescent probes conjugated with photodynamic therapy agents can generate singlet oxygen under light activation to kill tumor cells [332] while simultaneously offering real‐time intraoperative imaging for precise delineation of tumor margins. Furthermore, multimodal platforms, such as the integration of PET/CT with fluorescence imaging, are being developed to facilitate both diagnosis and image‐guided treatment in patients with resectable malignant tumors.

To date, there have been no reported cases of targeted FAP fluorescence technology being applied to human IMI. However, several examples of its application in cancer animal models exist. Liu et al. [333] reported a highly sensitive fluorescent probe, cv‐fap, for detecting FAPα activity in melanoma, with a detection limit of 5.3 ng/mL. This probe exhibits a high binding affinity and overall catalytic efficiency, enabling it to effectively differentiate between normal cells in mice and malignant melanoma cells in tumor‐bearing mice [333]. In the case of fibrosarcoma, Tansi et al. [334] developed activatable FAP‐targeting immunoliposomes (FAP‐IL) for image‐guided detection of spontaneous metastases in mouse models. For HCC, a dual‐modal probe validated in both cellular and animal models of liver cancer has been mentioned [335]. Additionally, a fluorescent probe validated at both the cellular and animal levels using the MCF‐7 breast cancer cell line was also referenced in the context of breast cancer [148]. Dual‐modal probes combining PET/CT imaging and fluorescence imaging have gained increasing attention in tumor diagnosis and treatment research in recent years, with numerous preclinical studies emerging [336, 337, 338, 339]. To address the challenge of off‐target effects resulting from tumor heterogeneity, dual‐modal probes offer a promising solution. For partially resectable tumors, preoperative PET/CT screening to identify high‐expression populations, coupled with the selective use of fluorescence for intraoperative navigation, offers a feasible approach. This strategy may help overcome the clinical challenge of unstable imaging.

In summary, the imaging targets for CAFs in cancer diagnosis and treatment mainly focus on FAP, including PET/CT, fluorescence imaging, and other modalities. We have observed the promising potential of FAP‐targeted PET/CT in cancer diagnosis, which may serve as a viable alternative to FDG PET/CT for certain cancers. However, the lack of existing clinical trials prevents us from drawing definitive conclusions. Multicenter clinical trials with a larger number of cases are necessary. Fluorescence imaging is primarily aimed at resectable tumors, and the application of dual‐modal probes may facilitate the integration of tumor diagnosis and treatment, enhancing precision in both areas.

### 4.2 Imaging CAFs‐Related ECM

In addition to targeting specific biomarkers expressed by CAFs, targeting the dense ECM produced by CAFs represents another effective approach for tumor imaging. Molecular probes aimed at fibrotic components have been extensively studied in cardiac, hepatic, and pulmonary fibrosis, with several studies also reporting their application in tumor imaging [340, 341, 342, 343]. It is important to note that although many studies have validated the imaging capabilities of these probes in tumor animal models, no human trials have been reported to date. The lack of specificity, due to the widespread presence of collagen in normal tissues, is likely a major limiting factor. Therefore, further refinement of their application scenarios is necessary.

Dynamic contrast‐enhanced magnetic resonance imaging (DCE‐MRI) is an imaging technique that involves the intravenous administration of contrast agents, typically gadolinium‐based, followed by continuous image acquisition. This method enables quantitative or semi‐quantitative analysis of the microvascular characteristics of tumor tissues and plays a significant role in tumor diagnosis, grading, treatment response assessment, and prognosis prediction [344, 345, 346, 347, 348]. In 2017, Polasek et al. [349] developed a magnetic resonance (MR) probe targeting type I collagen and validated its efficacy in a mouse model of pancreatic cancer. Farace et al. [350] evaluated the effectiveness of two DCE‐MRI probes in mouse models of prostate and pancreatic cancers, confirming the feasibility of targeted stromal imaging. In 2023, a preclinical study reported the use of a MR probe targeting type I collagen to detect lesions in a mouse model of endometrial cancer [351]. The success of preclinical studies does not necessarily translate to clinical applicability for collagen‐targeted imaging. Its major limitation, a lack of specificity, raises concerns about potential imaging artifacts and reduced diagnostic accuracy. Nevertheless, collagen‐targeted probes have shown notable potential in predicting the efficacy of neoadjuvant therapy, highlighting a promising avenue for further investigation. Following neoadjuvant therapy for various cancers, both the lesion and the surrounding tissue typically exhibit increased fibrosis [352, 353]. Erstad et al. [354] applied a type I collagen‐targeted MR probe to predict the prognosis of patients with pancreatic cancer following neoadjuvant therapy. Their findings indicated that tumor fibrosis after chemoradiotherapy correlates with OS and disease‐free survival [354].

PET probes targeting collagen have shown significant clinical translation potential, particularly in the study of cardiac and pulmonary fibrosis. CBP8, a PET probe targeting collagen, has already entered clinical trials for pancreatic cancer to assess its efficacy (NCT04485286). Furthermore, it has demonstrated favorable biological metabolism in the human body. Studies have indicated that CBP8 PET/MRI can predict treatment responses in pancreatic cancer patients [355, 356, 357]. Although no other studies have been reported thus far, the results of the ongoing CBP8 clinical trials are highly anticipated.

Integrins are heterodimeric transmembrane receptors composed of α and β subunits. They play a critical role in tumor initiation, progression, metastasis, and immune evasion by mediating interactions between cells and the ECM, as well as facilitating cell– communication [358, 359, 360, 361]. Integrin αvβ6 is a distinct heterodimer within the integrin family, consisting of αv and β6 subunits, and is characterized by unique biological functions and tumor‐associated properties. Its expression is significantly upregulated in certain malignant tumors, making it a promising target for molecular imaging [362, 363]. Kimura et al. [364] developed and validated a novel engineered cystine knot peptide (knottin) that selectively binds to human integrin αvβ6 with sub‐nanomolar affinity. The study demonstrated its effectiveness in detecting multiple cancers, including pancreatic, cervical, and lung cancers, in patients across two research sites [364].

Compared with FAP, targeting integrins and collagen presents a natural disadvantage due to their strong nonspecific expression, which poses a key obstacle to clinical translation. Additionally, the absence of certain stromal components in some cancers can lead to suboptimal imaging performance. A potential solution is to focus on cancers with high fibrous content, such as pancreatic cancer, rather than pursuing pan‐cancer approaches. FAPI targeting FAP, along with new tracers, deserves more attention, particularly given FAP's role as a specific marker of CAFs. Further clinical trials are essential to confirm imaging efficacy in specific cancer types.

## 5 Therapeutic Targeting of CAFs

The increasing understanding of the TME has shifted the focus beyond cancer cells as the sole target. CAFs, as prevalent stromal cells, have been extensively studied in recent years, leading to the development of numerous drugs targeting them. Several comprehensive reviews have classified these drugs from various perspectives [365, 366, 367]. This paper highlights landmark clinical trials and emphasizes the clinical translational potential of targeting CAFs. Approaches to targeting CAFs can be broadly classified into two categories: direct targeting and indirect targeting. Recent research on reprogramming CAFs has also emerged. Direct targeting involves using drugs to target specific markers on CAFs, resulting in their depletion. In contrast, indirect targeting focuses on key regulatory pathways in CAFs, particularly the TGF‐β and CXCL12/CXCR4 axes. Current clinical trials targeting CAFs are summarized in Table 2.

**TABLE 2:** Clinical application of CAFs in cancer therapy.

Clinical trial number(s)	Research title	Stage	Target	Probe	Treatment modality	Cancer types	Outcomes
NCT04939610	LuMIERE: A phase 1/2, multicenter, open‐label, non‐randomized study to investigate safety and tolerability, pharmacokinetics, dosimetry, and preliminary activity of ^177^Lu‐FAP‐2286 in patients with an advanced solid tumor	II	FAP	^177^Lu–FAP‐2286	Targeted radionuclide therapy	Solid tumor	Administration of ^177^Lu–FAP‐2286 was well tolerated, with no adverse symptoms or clinically detectable pharmacologic effects reported in any of the patients. Significant uptake and prolonged tumor retention of ^177^Lu–FAP‐2286 resulted in high absorbed tumor doses.
			FAP	^177^Lu–FAP‐2286	Targeted radionuclide therapy	Advanced lung cancer	Status scores indicated that the overall health status, symptom response, and quality of life of patients improved after treatment with ^177^Lu–FAP‐2286.
NCT05410821	Evaluation of ^177^Lu‐DOTA‐EB‐FAPI in patients with metastatic radioactive iodine refractory thyroid cancer	I	FAP	^177^Lu–DOTA–EB–FAPI	Targeted radionuclide therapy	Refractory thyroid gland papillary carcinoma	FAP‐targeted radioligand therapy with ^177^Lu–LNC1004 at 3.33 GBq per cycle was well tolerated in patients with advanced mRAIR–TC, delivering a high radiation dose to tumor lesions, which encouraged therapeutic efficacy while maintaining acceptable side effects.
NCT02627274	An open‐label, multicenter, dose‐escalation, phase Ia/Ib study to evaluate safety, pharmacokinetics, and therapeutic activity of RO6874281, an immunocytokine consisting of interleukin 2 variant (IL‐2v) targeting fibroblast activation protein‐α (FAP), as a single agent (Part A) or in combination with trastuzumab or cetuximab (part B or C)	I	FAP	RO6874281	Study drug administration	Breast cancer, cancer of the head and neck	FAP–IL2v demonstrated a manageable safety profile and showed initial signs of antitumor activity in patients with advanced or metastatic solid tumors.
							The safety profile of FAP–IL2v in combination with cetuximab was found to be acceptable.
NCT03386721	An open‐label, multicenter, phase II study to evaluate the therapeutic activity of simlukafusp alfa (RO6874281), an immunocytokine, consisting of interleukin‐2 variant (IL‐2v) targeting fibroblast activation protein‐Α (FAP), in combination with atezolizumab (anti‐PD‐L1), administered intravenously, in participants with advanced and/or metastatic solid tumors	II	FAP	RO6874281	Study drug administration	Advanced/metastatic head and neck, esophageal, and cervical cancers	FAP–IL2v combined with atezolizumab demonstrated clinical activity and manageable safety in patients with recurrent and/or metastatic cervical SCC.
NCT01722149	Phase I study for the adoptive transfer of re‐directed FAP‐specific T cells in the pleural effusion of patients with malignant pleural mesothelioma	I	FAP	Redirected T cells	Study drug administration	Malignant pleural mesothelioma	When injected into the pleural effusion of a patient with malignant pleural mesothelioma (MPM), the Δ‐CD28 CAR could be detected for up to 21 days and exhibited functionality.
NCT03910660	A trial of BXCL701 and pembrolizumab in patients with mCRPC either small cell neuroendocrine prostate cancer or adenocarcinoma phenotype	II	FAP	BXCL701	Study drug administration	Prostate cancer, neuroendocrine tumors, and small cell carcinoma	BXCL701 in combination with pembrolizumab showed antitumor effects in neuroendocrine prostate cancer, with all responders being patients with low microsatellite stability (MSS) and/or tumor mutational burden (TMB), while maintaining a controllable safety profile.
NCT03277209	To assess the safety of continuous IV administration of plerixafor and assess impact on the immune microenvironment in patients with pancreatic, ovarian and colorectal adenocarcinomas	I	CXCR4	Plerixafor	Study drug administration	Pancreatic cancer	The study was terminated due to slow accrual.
NCT02826486	Study assessing safety and efficacy of combination of BL‐8040 and pembrolizumab in metastatic pancreatic cancer patients (COMBAT)	II	CXCR4	BL‐8040	Study drug administration	Metastatic pancreatic cancer	A total of 43 patients were enrolled. The overall response rate (ORR) according to RECIST v1.1 was 21.1%, with a confirmed ORR of 13.2%. The disease control rate (DCR) was 63.2%, with a median duration of clinical benefit of 5.7 months. In the intention‐to‐treat population, the median progression‐free survival (PFS) was 3.8 months, and the median overall survival (OS) was 6.6 months. The triple combination was safe and well tolerated, with toxicity comparable to the NAPOLI‐1 regimen. Notably, the incidence of grade 3 or higher neutropenia and infection was 7%, which is lower than expected for this chemotherapy regimen.
NCT01837095	Dose escalation of POL6326 in combination with eribulin in patients with metastatic breast cancer	I	CXCR4	POL6326	Study drug administration	Metastatic breast cancer	The safety and tolerability of balixafortide plus eribulin appear similar to those of eribulin or balixafortide monotherapy. The preliminary activity of this combination seems promising in patients with HER‐negative metastatic breast cancer, suggesting potential as a new therapeutic option for heavily pretreated patients and warranting further investigation in randomized trials.
NCT03095781	Pembrolizumab and XL888 in patients with advanced gastrointestinal cancer	I	HSP90	XL888	Study drug administration	Advanced gastrointestinal cancer	Among the 16 patients receiving treatment, no objective responses were observed; however, four patients (25%) achieved stable disease. The median PFS was 1.9 months, and the median OS was 5.5 months.
NCT01401062	Fresolimumab and radiotherapy in metastatic breast cancer	II	TGF‐β	Fresolimumab	Study drug administration	Metastatic breast cancer	Preliminary data support the rationale for investigating the potential benefits of adding PD‐1 blockade to enhance responses to TGFβ blockade and radiotherapy.
							Tumor‐suppressive effects of TGF‐β persist in some breast cancer patients at the time of surgery and can impact clinical outcomes.
NCT02937272	A study of LY3200882 in participants with solid tumors	I	TGF‐β	LY3200882	Study drug administration	Solid tumors	In treatment‐naïve patients with advanced pancreatic cancer, six of 12 patients achieved partial responses (PR) according to Response Evaluation Criteria in Solid Tumors (RECIST) v1.1, and three of 12 patients demonstrated stable disease, resulting in an overall disease‐control rate of 75% with the combination of LY3200882, gemcitabine, and nab‐paclitaxel.
NCT03620201	M7824 in treating patients with stage II‐III HER2 positive breast cancer	I	PD‐L1 and TGFβ	M7824	Study drug administration	StageII–III HER2‐positive breast cancer	
NCT02517398	MSB0011359C (M7824) in metastatic or locally advanced solid tumors	I	PD‐L1 and TGFβ	M7824	Study drug administration	Metastatic or locally advanced solid tumors	M7824 has a manageable safety profile in patients with heavily pretreated advanced solid tumors.
							Treatment‐related adverse events occurred in 55 of the 80 patients (69%), with 23 of the 80 patients (29%) experiencing grade 3 or higher events.
NCT03493945	Phase I/​II study of immunotherapy combination BN‐brachyury vaccine, M7824, N‐803 and epacadostat (QuEST1)	II	PD‐L1 and TGFβ	M7824	Study drug administration	Metastatic prostate cancer	The immune effects of tumor‐directed vaccines, PD‐L1 blockade, TGF‐β sequestration, IL‐15 agonism, and IDO1 inhibition can be additive and/or synergistic. This includes TGF‐β’s putative role in T‐cell exclusion from the tumor microenvironment (TME) in metastatic urothelial carcinoma, which can be reversed with dual targeting of TGF‐β and PD‐L1.
NCT02699515	MSB0011359C (M7824) in participants with metastatic or locally advanced solid tumors	I	PD‐L1 and TGFβ	M7824	Study drug administration	Metastatic or locally advanced solid tumors	M7824 demonstrated evidence of clinical activity with prolonged objective responses and maintained a manageable safety profile.
NCT03192345	A first‐in‐human study of the safety, pharmacokinetics, pharmacodynamics and anti‐tumor activity of SAR439459 monotherapy and combination of SAR439459 and cemiplimab in patients with advanced solid tumors	I	PD‐L1 and TGFβ	SAR439459	Study drug administration	Malignant solid tumor	SAR’459, both alone and in combination with cemiplimab, exhibited a noteworthy safety profile, and the maximum tolerated dose (MTD) was not reached during the dose‐escalation phase. However, due to insufficient antitumor responses and observed bleeding risks, particularly in the hepatocellular carcinoma (HCC) cohort, the study was terminated during the expansion phase, and the antitumor activity of SAR’459 was not further investigated.
NCT04729725	SAR439459 and cemiplimab for the treatment of advanced or unresectable solid tumors, strategic alliance, TACTIC TRIAL	I	PD‐L1 and TGFβ	SAR439459	Study drug administration	Advanced or unresectable solid tumors	SAR439459 has shown promising responses in combination with cemiplimab in some patients refractory to immune checkpoint inhibitors; however, further studies are needed to identify biomarkers of response.
NCT04064190	Vactosertib with durvalumab in urothelial carcinoma failing checkpoint inhibition	II	PD‐L1 and TGFβ	Vactosertib (TEW‐7197)	Study drug administration	Urothelial carcinoma	Withdrawn
NCT03802084	A study to evaluate the safety and efficacy of vactosertib and imatinib in patients with advanced desmoid tumor	II	PD‐L1 and TGFβ	Vactosertib (TEW‐7197)	Study drug administration	Desmoid tumor	The combination of vactosertib and imatinib was well tolerated and showed promising clinical activity in patients with progressive, locally advanced desmoid tumors.
NCT03143985	Vactosertib in combination w/​ pomalidomide in relapsed or relapsed and refractory multiple myeloma	I	PD‐L1 and TGFβ	Vactosertib (TEW‐7197)	Study drug administration	Relapsed or relapsed and refractory multiple myeloma	Vactosertib is a safe therapeutic that demonstrates tumor‐intrinsic activity and can overcome immunosuppressive challenges within the tumor microenvironment to reinvigorate T‐cell fitness.
NCT02160106	First in human dose escalation study of vactosertib (TEW‐7197) in subjects with advanced stage solid tumors vactosertib	I	PD‐L1 and TGFβ	Vactosertib (TEW‐7197)	Study drug administration	Advanced stage solid tumors	Vactosertib's pharmacokinetics were dose‐proportional within the tested dose range, with negligible accumulation when administered once daily for five days.
NCT02423343	A study of galunisertib (LY2157299) in combination with nivolumab in advanced refractory solid tumors and in recurrent or refractory NSCLC, or hepatocellular carcinoma	II	PD‐L1 and TGFβ	Galunisertib (LY2157299)	Study drug administration	Solid tumor, recurrent non‐small cell lung cancer, recurrent hepatocellular carcinoma	The study met its primary endpoint, as galunisertib combined with nivolumab was well tolerated. Preliminary efficacy was observed in a subset of patients in the phase 2 non‐small cell lung cancer (NSCLC) cohort.
NCT02688712	ExIST study of LY2157299 (galunisertib) in rectal cancer	II	PD‐L1 and TGFβ	Galunisertib (LY2157299)	Study drug administration	Rectal adenocarcinoma	The addition of galunisertib to neoadjuvant chemoradiotherapy in patients with locally advanced rectal cancer improved the complete response rate to 32%, was well tolerated, and warrants further assessment in randomized trials.
NCT02734160	A study of galunisertib (LY2157299) and durvalumab (MEDI4736) in participants with metastatic pancreatic cancer	I	PD‐L1 and TGFβ	Galunisertib (LY2157299)	Study drug administration	Metastatic pancreatic cancer	Galunisertib 150 mg twice daily, coadministered with durvalumab 1500 mg every 4 weeks, was tolerable.
NCT02508532	(NAVIGATOR) Study of BLU‐285 in patients with gastrointestinal stromal tumors (GIST) and other relapsed and refractory solid tumors	I	PDGFR	Avapritinib	Study drug administration	GIST, other relapsed or refractory solid tumors	Avapritinib showed clinical activity against PDGFRA D842V‐mutant and later‐line KIT‐mutant gastrointestinal stromal tumors (GIST).
NCT03465722	(VOYAGER) Study of avapritinib vs regorafenib in patients with locally advanced unresectable or metastatic GIST	III	PDGFR	Avapritinib	Study drug administration	Locally advanced unresectable or metastatic GIST	The primary endpoint was not met. There was no significant difference in median PFS between avapritinib and regorafenib in patients with molecularly unselected, late‐line GIST.
NCT02856425	Trial of pembrolizumab and nintedanib (PEMBIB)	I	FGFR, PDGFR, and VEGFR	Nintedanib	Study drug administration	Advanced solid tumors	Nintedanib 150 mg bid is the recommended dose for combination with pembrolizumab and is currently being investigated in multiple expansion cohorts.
NCT02699606	A study to evaluate the clinical efficacy of JNJ‐42756493 (erdafitinib), a pan‐fibroblast growth factor receptor (FGFR) tyrosine kinase inhibitor, in asian participants with advanced non‐small‐cell lung cancer, urothelial cancer, esophageal cancer or cholangiocarcinoma	II	FGFR	Erdafitinib	Study drug administration	Neoplasm	All patients experienced treatment‐emergent adverse events, with grade ≥3 treatment‐emergent adverse events reported in 22 (62.9%) patients. Hyperphosphatemia was the most frequently reported treatment‐emergent adverse event (all‐grade, 85.7%).
NCT03390504	A study of erdafitinib compared with vinflunine or docetaxel or pembrolizumab in participants with advanced urothelial cancer and selected fibroblast growth factor receptor (FGFR) gene aberrations (THOR)	III	FGFR	Erdafitinib	Study drug administration	Urothelial cancer	Erdafitinib therapy resulted in significantly longer overall survival compared to chemotherapy among patients with metastatic urothelial carcinoma and FGFR alterations after previous anti‐PD‐1 or anti‐PD‐L1 treatment.
NCT04083976	A study of erdafitinib in participants with advanced solid tumors and fibroblast growth factor receptor (FGFR) gene alterations (RAGNAR)	II	FGFR	Erdafitinib	Study drug administration	Advanced solid tumor	At a median follow‐up of 17.9 months (IQR 13.6–23.9), an objective response was observed in 64 (30% [95% CI 24–36]) of 217 patients across 16 distinct tumor types.
NCT02365597	An efficacy and safety study of erdafitinib (JNJ‐42756493) in participants with urothelial cancer	II	FGFR	Erdafitinib	Study drug administration	Urothelial cancer	The use of erdafitinib was associated with an objective tumor response in 40% of previously treated patients with locally advanced and unresectable or metastatic urothelial carcinoma harboring FGFR alterations.
NCT04172675	A study of erdafitinib versus investigator choice of intravesical chemotherapy in participants who received Bacillus Calmette‐Guérin (BCG) and recurred with high risk non‐muscle‐invasive bladder cancer (NMIBC)	II	FGFR	Erdafitinib	Study drug administration	Urinary bladder neoplasms	Seventy‐three patients were randomized in a 2:1 ratio to receive erdafitinib (*n* = 49) or chemotherapy (*n* = 24). Median follow‐up for recurrence‐free survival (RFS) was 13.4 months for both groups. Median RFS was not reached for erdafitinib [95% CI 16.9 months–not estimable], while it was 11.6 months (95% CI 6.4–20.1 months) for chemotherapy, with an estimated hazard ratio of 0.28 (95% CI 0.1–0.6; nominal *p* value = 0.0008).
NCT04241276	A randomised trial of ATRA in a novel drug combination for pancreatic cancer (STARPAC2)	II	Vitamin A	ATRA	Study drug administration	Pancreatic cancer	Active, not recruiting
NCT03307148	A phase 1B study repurposing ATRA as stromal targeting agent along with gemcitabine and nab‐paclitaxel for pancreatic cancer (STAR_PAC)	I	Vitamin A	ATRA	Study drug administration	Pancreatic cancer	The simulations suggest that the STARPAC design performed well in estimating the maximum tolerated dose (MTD) and in treating patients at the highest possible therapeutic levels. STARPAC and TITE‐CRM were comparable in terms of the number of patients required and dose‐limiting toxicities (DLTs) incurred.
NCT03520790	Vitamin D receptor agonist paricalcitol plus gemcitabine and nab‐paclitaxel in patients with metastatic pancreatic cancer	I	Vitamin D	Paricalcitol	Study drug administration	Pancreatic cancer	Terminated
NCT03331562	A SU2C Catalyst® trial of a PD1 inhibitor with or without a vitamin D analog for the maintenance of pancreatic cancer	I	Vitamin D	Paricalcitol	Study drug administration	Pancreatic cancer	Paricalcitol did not improve the efficacy of pembrolizumab, likely due to its short half‐life of only 5–7 h. Microbiome analysis revealed significant differences between long‐term (>12 weeks) and short‐term (<12 weeks) survival groups across treatment arms. Modulation of the tumor microenvironment will likely require more sustained VDR activity.
NCT05064618	Phase I/II investigator‐initiated clinical trial of MIKE‐1 with gemcitabine and nab‐paclitaxel combination therapy for unresectable pancreatic cancer	II	Meflin	Am80	Study drug administration	Unresectable pancreatic cancer	

*Data source*: https://clinicaltrials.gov/.

*Abbreviations*: FAP: fibroblast activation protein, CXCR4: C‐X‐C chemokine receptor type 4, PDGFR: platelet‐derived growth factor receptor, TGF‐β: transforming growth factor‐beta, PD‐L1: programmed death‐ligand 1, HSP90: heat shock protein 90, HER2: human epidermal growth factor receptor 2, VEGFR: vascular endothelial growth factor receptor, mCRPC: metastatic castration‐resistant prostate cancer, MSS: microsatellite stability, TMB: tumor mutational burden, ORR: objective response rate, DCR: disease control rate, PFS: progression‐free survival, OS: overall survival, RECIST: response evaluation criteria in solid tumors, MTD: maximum tolerated dose, DLT: dose‐limiting toxicity, IDO1: indoleamine 2,3‐dioxygenase 1, TME: tumor microenvironment, BCG: Bacillus Calmette‐Guérin, RFS: relapse‐free survival, ATRA: all‐trans retinoic acid, VDR: vitamin D receptor, CAR: chimeric antigen receptor, SCC: squamous cell carcinoma, NSCLC: non‐small cell lung cancer, HCC: hepatocellular carcinoma, Q4W: every 4 weeks, bid: twice daily, FGFR: fibroblast growth factor receptor, GIST: gastrointestinal stromal tumor.

### 5.1 Targeting Signaling Pathways in CAFs

Several key mechanisms and pathways involved in the activation of CAFs, the promotion of tumor proliferation and metastasis, and the establishment of an immunosuppressive microenvironment have been discussed in previous sections. Targeted drugs have been developed and extensively studied to inhibit these pathways, each with distinct characteristics and key molecules. The most closely monitored drug types are those targeting the TGF‐β pathway and the CXCL12/CXCR4 axis [368, 369, 370]. Numerous influential clinical studies have demonstrated the therapeutic potential of these drugs, particularly when used in combination with immunotherapies such as anti‐PD‐1/PD‐L1 [371, 372].

#### 5.1.1 Transforming Growth Factor‐β

Blocking TGF‐β is currently the most extensively studied therapeutic approach targeting CAFs. Mechanistic studies have highlighted the immunosuppressive role of TGF‐β within the TME, making its combination with PD‐1/PD‐L1 blockade a promising therapeutic strategy. Additionally, TGF‐β signaling is a crucial regulator of CAF maturation. Inhibiting this pathway holds promise for normalizing CAFs or inducing a dormant state, potentially mitigating their tumor‐promoting effects [373].

LY2157299 (galunisertib) is a small molecule inhibitor of TGF‐β that blocks CAF activation and suppresses its immunosuppressive effects. This drug has been evaluated for efficacy and safety in various cancers, including liver cancer and malignant glioma, both as a monotherapy and in combination with other treatments. However, the results have been mixed, with varying therapeutic effects [374, 375, 376, 377, 378, 379, 380, 381, 382, 383, 384, 385, 386, 387, 388]. Ongoing clinical trials are currently assessing its potential in lung, liver, and pancreatic cancers. In response to the drug resistance associated with TGF‐β application in pancreatic cancer, some studies have suggested that autocrine chemokines secreted by CAFs can induce their transformation into an inflammatory phenotype. These inflammatory phenotype CAFs promote treatment resistance through paracrine signaling, further complicating therapeutic strategies [389]. LY3200882 is a TGF‐β receptor inhibitor that has been tested in clinical trials for various advanced cancers [390, 391]. Studies have demonstrated that its combination with immunotherapy can enhance its therapeutic effect in TNBC [392]. Additionally, LY3200882 promotes the release of interferon‐β by macrophages, thereby improving the efficacy of radiotherapy in tumor‐bearing mice with head and neck cancer and lung cancer [393]. Vactosertib is an ALK5 antagonist targeting the TGF‐β receptor. Several studies are currently underway to explore its therapeutic potential. Notably, it has shown promise in the treatment of desmoid tumors when combined with imatinib [394, 395, 396].

#### 5.1.2 CXCL12/CXCR4 and CSF1

CAFs are considered the primary source of CXCL12, which exerts an immunosuppressive effect by binding to its receptor, CXCR4 [397]. Targeting the CXCL12/CXCR4 pathway derived from CAFs has been shown to be effective in mouse models of pancreatic and CRCs, leading to increased T‐cell infiltration [398]. Similar results were observed in targeted therapies that antagonize CSF1/CSF1R signaling, indicating the potential for macrophage reprogramming [399, 400]. Studies in melanoma have also demonstrated that targeting CXCR4 results in a reduction of myeloid cells and the activation of NK cells [401]. BL‐8040 is a CXCR4 antagonist that has shown therapeutic potential in pancreatic cancer when combined with pembrolizumab and chemotherapy. In healthy individuals, it can rapidly recruit CD34+ cells [402, 403]. AMD3100 is another CXCR4 antagonist with a mechanism of action similar to that of BL‐8040. It is used in the treatment of various diseases, including but not limited to cancer. In multiple myeloma and lymphoma, AMD3100 has demonstrated notable therapeutic efficacy [404, 405, 406, 407, 408, 409, 410]. The primary function of this class of drugs is to enhance stem cell mobilization and promote T‐cell infiltration within the TME.

#### 5.1.3 Other Pathways

IL‐6 is a key immunosuppressive factor secreted by iCAFs. IL‐6 antagonists have been widely used in the treatment of autoimmune diseases. In cancer therapy, their combination with PD‐L1 blockade has shown therapeutic efficacy in mouse models of melanoma and pancreatic cancer by disrupting the immunomodulatory interactions mediated by IL‐6 [411, 412]. IL‐6 secreted by CAFs can increase the levels of TIMP‐1 in the extracellular environment. TIMP‐1, in turn, activates the STAT3 signaling pathway, which is considered a key molecular mechanism promoting the proliferation and invasion of breast cancer cells [413, 414].

The Hedgehog (Hh) signaling pathway plays a critical role in the proliferation and invasion of various tumors. While largely inactive in normal adult tissues, it becomes reactivated within the TME [415, 416, 417]. CAFs can stimulate Hh signaling through paracrine mechanisms [182, 418]. Smoothened (SMO), a key transmembrane protein in the Hh pathway, mediates this activation, leading to aberrant expression of downstream Gli transcription factors [419, 420]. Several SMO inhibitors have been approved and have demonstrated clinical efficacy in tumors such as basal cell carcinoma [421, 422, 423, 424]. A representative drug is vismodegib (GDC‐0449), a first‐in‐class small molecule inhibitor targeting the Hh signaling pathway. In a phase I clinical trial, vismodegib demonstrated a response rate of 58% in patients with advanced basal cell carcinoma, highlighting its therapeutic potential [402]. Gli is the central effector molecule of the Hh signaling pathway. Direct inhibition of Gli activity can effectively disrupt the maintenance of tumor stemness and suppress metastatic potential [425, 426]. GANT61 has shown therapeutic efficacy in osteosarcoma, ovarian cancer, and lung cancer by inhibiting the transcriptional activity of Gli, thereby suppressing tumor growth and progression [427, 428, 429]. Hh acyltransferase (Hhat), the enzyme responsible for palmitoylating Sonic hedgehog (Shh), has emerged as a novel therapeutic target for inhibiting Shh signaling in pancreatic cancer cells. By blocking Hhat activity, the posttranslational modification and subsequent signaling of Shh can be disrupted, potentially attenuating tumor growth and progression [430, 431]. RU‐SKI 43, a small‐molecule inhibitor of Hhat, has demonstrated potential therapeutic efficacy in preclinical models of pancreatic cancer, lung cancer, and breast cancer. By blocking the palmitoylation of Shh, RU‐SKI 43 effectively disrupts downstream Hh signaling, thereby impairing tumor growth and progression in these cancer types [432, 433, 434].

Targeting key molecules within CAF‐associated signaling pathways has produced promising therapeutic outcomes, particularly when combined with ICIs, as this strategy can effectively mitigate immune suppression within the TME. However, due to tumor heterogeneity, a universal or broad‐spectrum therapeutic approach has yet to be established. Identifying patient populations and tumor types most likely to benefit from such treatments remains a crucial area for further investigation. In conclusion, evidence from numerous clinical studies supports the viability of targeting CAF‐related pathways as a strategy in cancer therapy.

### 5.2 Targeting CAFs’ Specific Biomarkers

One extensively studied therapeutic strategy for targeting CAFs involves focusing on their specific biomarkers. FAP and α‐SMA are two of the most prominent targets under investigation. Therapeutic modalities developed against these markers include monoclonal antibodies, small‐molecule inhibitors, radionuclide therapies, chimeric antigen receptor T (CAR‐T) cell therapies, and novel vaccines. However, the antitumor efficacy of depleting FAP⁺ CAFs remains uncertain. In pancreatic cancer, for instance, the ablation of FAP⁺ CAFs has paradoxically led to accelerated tumor progression and increased Treg cell infiltration, raising concerns about the safety and effectiveness of directly targeting FAP and α‐SMA [435, 436]. These contradictory outcomes highlight the need for a deeper understanding of the underlying mechanisms.

#### 5.2.1 Fibroblast Activation Protein

Eliminating FAP‐positive CAFs (FAP⁺ CAFs) has become a key focus in the development of CAF‐targeted therapeutic strategies. Since FAP is a membrane protein specifically expressed by CAFs and is involved in nearly all tumor‐promoting processes, targeting FAP represents a promising approach to disrupt the protumorigenic functions of the tumor stroma [437, 438, 439, 440]. Various therapeutic strategies targeting FAP are currently under investigation, including CAR‐T cell therapy, monoclonal antibody therapy, vaccines, and radionuclide‐based treatments.

The use of antibodies to eliminate FAP+ CAFs was one of the earliest attempts, primarily aimed at enhancing antitumor immunity. It is important to note that targeting FAP to eliminate the tumor stroma does not inherently lead to antitumor effects. In mouse models of pancreatic cancer, the ablation of FAP⁺α‐SMA⁺ CAFs paradoxically resulted in accelerated tumor progression and increased infiltration of Treg cells [436]. The therapeutic efficacy of FAP‐targeted antibody monotherapy has not met expectations [441, 442]. This class of agents is primarily represented by the monoclonal antibody F19 and its humanized derivative, sibrotuzumab. Existing studies on sibrotuzumab have primarily focused on evaluating its safety profile, while its therapeutic efficacy has been unsatisfactory [443]. These findings suggest that, although FAP antibodies demonstrate tumor accumulation, their cytotoxic efficacy against tumor cells is limited. Recent studies indicate that conjugating FAP antibodies with immunotoxins may provide a promising strategy to enhance their therapeutic potential. In mouse models of breast cancer and melanoma, FAP antibody‐conjugated immunotoxins have exhibited robust antitumor efficacy. However, to date, no clinical trials in humans have been reported, and further research is needed to evaluate their safety and therapeutic potential in clinical settings [444, 445]. Compared with the use of antibodies targeting FAP alone, antibody‐conjugated radionuclide therapy and enzyme inhibitor‐conjugated radionuclide therapy have shown more promising potential in both preclinical and clinical trials. These approaches offer enhanced therapeutic efficacy and could represent more effective strategies for targeting CAFs.

TRT for FAP, a subspecialty within nuclear medicine, is rapidly advancing. As a pan‐cancer target, FAP holds significant potential and should not be overlooked in the development of precision cancer therapies. The earliest achievement in FAP‐TRT was the development of the ¹^3^¹I‐labeled monoclonal antibody F19. Several FAP‐TRTs are currently being applied to various cancer types, including pancreatic cancer, lung cancer, and gastrointestinal tumors. Covalent targeted radioligand (CTR) represents a novel drug modality that selectively binds radioactive ligands to tumors. This technology enhances tumor uptake and retention of radioligands while minimizing their absorption in circulation and healthy tissues. Cui et al. [446] developed an innovative CTR targeting FAP by incorporating a latent warhead into the ligand. This modification enables covalent bonding to the target protein without compromising affinity, resulting in the irreversible modification of the chelating agent carrying the radionuclide to the target. Upon reaching the tumor, the CTR first binds noncovalently to the target, then accelerates covalent (irreversible) bonding via the proximity effect, potentially enhancing affinity and reducing the tumor's drug clearance rate. Unbound CTR is rapidly excreted from the body. In mice with subcutaneous tumors, the drug demonstrated promising tumor control efficacy [446].

In pancreatic cancer, multiple FAP‐targeted probes, such as TEFAPI‐06 and 07, as well as ^177^Lu–FAPI‐46, have demonstrated improved tumor retention and significant tumor growth inhibition in mouse models. Clinical trials using ^90^Y–FAPI‐46 in pancreatic cancer have shown disease stabilization, while ^177^Lu–FAPI‐46 has exhibited favorable targeting and pharmacokinetics [447, 448, 449, 450, 451, 452, 453]. In thyroid cancer, ^177^Lu–EB–FAPI has resulted in partial remission and disease stabilization in patients with metastatic disease [331, 454, 455]. Studies on sarcoma [451, 453, 456, 457] and glioblastoma [330, 458, 459] also report promising results with FAP‐targeted therapies, though adverse events such as hematological toxicity have been observed. Other cancers, including lung and breast cancers, have also shown positive responses to FAP‐based TRT, with some patients experiencing significant disease control and reduced metastasis [460, 461, 462, 463, 464, 465, 466, 467]. The combined use of TRT and ICIs presents promising therapeutic potential. In preclinical models, treatment with ^177^Lu–LNC1004 has been shown to induce a transient upregulation of PD‐L1 expression on tumor cells, thereby sensitizing tumors to ICI therapy and enhancing overall antitumor efficacy [468]. However, the small sample sizes and limited clinical data highlight the need for further studies to establish the safety and efficacy of these treatments across larger cohorts.

CAR‐T cell therapy has been United States Food and Drug Administration‐approved for certain forms of lymphoma and leukemia, demonstrating significant success in hematological malignancies. However, its application in solid tumors remains challenging [469, 470, 471, 472]. One of the main obstacles is the identification and selection of specific tumor antigens that are consistently expressed across the TME. Unlike tumor cells, CAFs are genetically stable components of the TME, making them an attractive target for CAR‐T cell therapy. Several preclinical studies have demonstrated the feasibility of CAR‐T cell therapy targeting FAP, but the results of clinical trials are still pending. Addressing the considerable heterogeneity in solid tumors poses a significant challenge [473, 474]. A phase I clinical trial of CAR‐T cell therapy targeting FAP was conducted in patients with malignant pleural mesothelioma. The results indicated promising safety, with two‐thirds of the patients surviving the treatment [475]. Two additional studies are underway, and their results are worth monitoring (NCT03932565, NCT03198546). However, the path to effectively implementing CAR‐T cell therapy targeting FAP in broader clinical applications remains challenging and requires further development and research.

Tumor vaccines represent an emerging therapeutic approach in oncology, designed to activate antigen‐presenting cells and T lymphocytes, which play a crucial role in initiating the cancer immune cycle. By stimulating the immune system, tumor vaccines are considered one of the most promising therapies for enhancing antitumor immunity [476, 477, 478, 479]. Currently, both DNA vaccines and DC vaccines targeting FAP are in the preclinical research stage. In animal models, these vaccines have demonstrated strong CD8⁺ T cell activation, high targeting specificity, and promising tumor‐suppressive effects [480, 481, 482]. Due to the lack of clinical trials, the efficacy and safety of FAP‐targeted vaccines cannot yet be confirmed. Further validation through human studies is essential to evaluate their clinical potential.

#### 5.2.2 α‐Smooth Muscle Actin

α‐SMA, another marker of CAFs, is considered a potential biomarker in HER2‐positive breast cancer [483]. However, research targeting α‐SMA remains in the preclinical stage, with contradictory results observed in mouse models. On one hand, in a mouse model of breast cancer, docetaxel‐conjugated nanoparticles targeting α‐SMA‐expressing stromal cells effectively inhibited metastasis, demonstrating potential therapeutic efficacy [484]. On the other hand, the targeted elimination of FAP+α‐SMA+ CAFs leads to enhanced tumor stemness and increased infiltration of Treg cells, accelerating tumor progression [435]. It is important to note that α‐SMA, as a marker of smooth muscle cells, is widely expressed in normal tissues such as blood vessels and the digestive tract [485, 486, 487]. This broad expression raises significant toxicity concerns in in vivo experiments. Although current organ‐specific delivery strategies may offer potential improvements [488, 489, 490, 491], the efficacy and safety of α‐SMA‐targeted therapies in humans remain unsatisfactory. Overall, the therapeutic application of α‐SMA targeting across various cancer types is viewed with skepticism.

### 5.3 Reprogramming CAFs

As an alternative to eliminating CAFs, converting activated CAFs into a quiescent state or reprogramming them into normal fibroblasts represents a promising therapeutic strategy. However, current research on this approach is primarily limited to PDAC mouse models. Future studies should aim to broaden its application to a wider range of preclinical cancer models and initiate clinical trials to assess its feasibility and therapeutic potential in humans.

**FIGURE 4:**
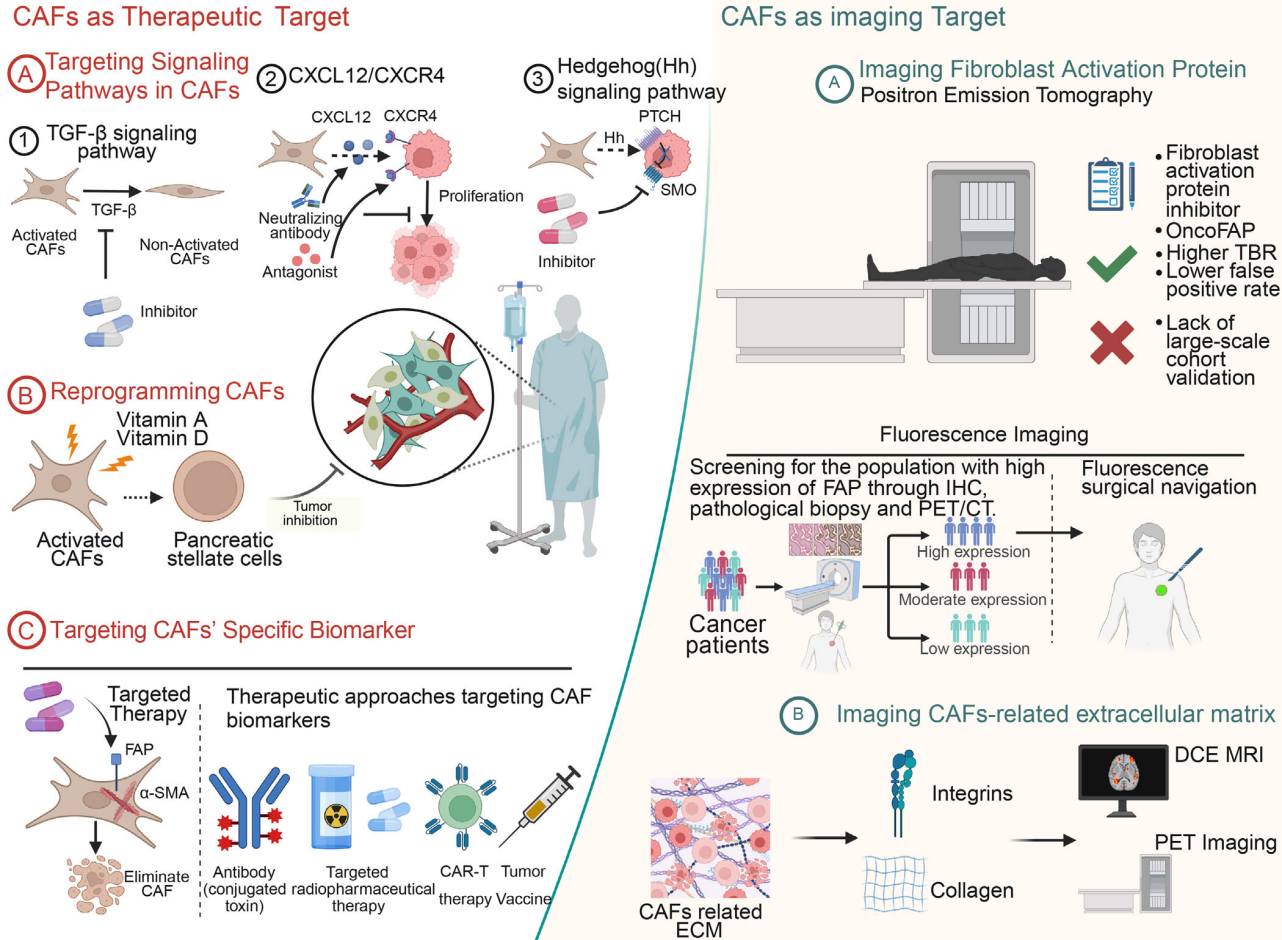
Overview of diagnosis and treatment strategies targeting cancer‐associated fibroblasts (CAFs). The left panel illustrates therapeutic approaches, including the inhibition of key signaling pathways such as TGF‐β and CXCL12/CXCR4, and Hedgehog using small‐molecule inhibitors and neutralizing antibodies to suppress CAF‐induced tumor progression. Reprogramming strategies aim to convert activated CAFs into quiescent states, such as pancreatic stellate cells, through agents like vitamins A or D, thereby reducing extracellular matrix (ECM) deposition and tumor‐supportive functions. Additionally, targeting specific CAF biomarkers, such as fibroblast activation protein (FAP) and alpha‐smooth muscle actin (α‐SMA), facilitates therapeutic interventions via monoclonal antibodies, radiopharmaceuticals, chimeric antigen receptor T (CAR‐T) cell therapy, and tumor vaccines. The right panel highlights diagnostic approaches that utilize CAFs as imaging targets. FAP‐based positron emission tomography (PET) imaging using fibroblast activation protein inhibitors (FAPI) offers a high tumor‐to‐background ratio and low false‐positive rates, though it requires further validation in large cohorts. Fluorescence imaging, informed by immunohistochemistry (IHC), biopsy, and PET/computed tomography (PET/CT), aids in identifying patients with high FAP expression for guided surgical navigation. Imaging of CAF‐derived ECM components, such as integrins and collagen, using dynamic contrast‐enhanced magnetic resonance imaging (DCE‐MRI) and PET, further enhances tumor visualization and assessment. *Abbreviations*: CAF, cancer‐associated fibroblast; TGF‐β, transforming growth factor‐beta; CXCL12, C‐X‐C motif chemokine ligand 12; CXCR4, C‐X‐C motif chemokine receptor 4; FAP, fibroblast activation protein; α‐SMA, alpha‐smooth muscle actin; CAR‐T, chimeric antigen receptor T cell; FAPI, fibroblast activation protein inhibitor; PET, positron emission tomography; IHC, immunohistochemistry; CT, computed tomography; ECM, extracellular matrix; DCE‐MRI, dynamic contrast‐enhanced magnetic resonance imaging (created in https://BioRender.com).

In PDAC, pancreatic stellate cells (PSCs) are the primary source of CAFs. Upon activation, PSCs differentiate into CAFs with myofibroblastic characteristics (myCAFs), leading to excessive ECM production [492, 493, 494, 495]. Treatment with all‐trans retinoic acid (ATRA) has been shown to induce quiescence in PSCs, thereby reducing paracrine Wnt/β‐catenin signaling and subsequently slowing the progression of PDAC [496]. When ATRA is combined with gemcitabine, it results in reduced proliferation and invasion of cancer cells while enhancing their apoptosis. Concurrently, the activity of PSCs, as indicated by the deposition of ECM proteins such as collagen and FN1, as well as their invasive capacity, is significantly decreased following the combination treatment [497]. A phase I clinical study involving patients with advanced unresectable PDAC demonstrated that repurposing ATRA as a stroma‐targeting agent in combination with gemcitabine and albumin‐bound paclitaxel is safe and well tolerated [498]. The mechanisms underlying CAF reprogramming in tumors other than PDAC remain under investigation, with no definitive evidence yet supporting reliable reprogramming pathways in these contexts. Nonetheless, the relatively successful reprogramming of CAFs in PDAC highlights the potential of this approach as a therapeutic strategy. Further mechanistic studies are essential to elucidate the key molecular pathways involved in CAF reprogramming and to facilitate the development of targeted therapeutic agents (Figure 4).

## 6 Conclusion and Future Directions

Advancements in our understanding of the TME have deepened insights into both tumor cells and the surrounding stromal components essential for their survival. Among these, CAFs play a pivotal role through complex paracrine signaling and diverse molecular pathways. As key contributors to ECM deposition, immune modulation, and the promotion of tumor invasion and metastasis, CAFs exhibit substantial heterogeneity, existing in multiple subtypes with distinct functional roles. The development of omics technologies, particularly single‐cell sequencing and spatial transcriptomics, has significantly enhanced our knowledge of the spatial and temporal diversity of CAFs within the TME. These studies highlight the variability of CAFs not only across different tumor types but also within individual tumors and among subtypes of the same tumor. Although numerous CAF‐specific markers have been identified, such as FAP and α‐SMA, current CAF classification remains broad, and future advancements in omics are expected to yield more specific markers, facilitating a better understanding of CAF heterogeneity, more precise prognostic assessments, and the development of targeted therapies. Importantly, research should increasingly focus on the heterogeneity of CAFs within individual tumors and pathological subtypes, rather than seeking universal markers, to avoid overlooking the intrinsic diversity of tumor biology.

The heterogeneity of CAFs reflects their diverse functions across different TMEs, with CAFs contributing to various tumorigenic processes, including tumor initiation, proliferation, invasion, metastasis, immune modulation, and therapeutic resistance. It is important to recognize that categorizing CAFs into the commonly referenced subtypes (myCAF, iCAF, and apCAF) may be overly simplistic, as functional overlap exists among these populations. In a cross‐tissue analysis of human fibroblasts using single‐cell sequencing, Gao et al. [499] identified distinct myofibroblast subtypes with varying roles in immune regulation, highlighting the need to move beyond traditional classifications and adopt a more nuanced understanding of fibroblast biology. It is essential to emphasize the previously underreported functions of CAFs, such as their role in remodeling the ECM to facilitate cancer cell migration, secreting EVs that promote metastatic colonization, and activating MSCs at metastatic sites to generate new CAFs. Simultaneously, attention should be given to the similarities and differences between primary tumor CAFs and those present at metastatic sites, as this knowledge could inform the development of more precise and broadly effective therapeutic strategies.

Current therapeutic strategies targeting CAFs primarily include the elimination of specific CAF phenotypes, indirect targeting of CAF‐associated molecular pathways and the tumor stroma, and reprogramming CAFs into a quiescent state. Traditional monoclonal antibodies against FAP have demonstrated limited therapeutic efficacy. Moreover, the ablation of FAP⁺α‐SMA⁺ CAFs in pancreatic cancer has paradoxically accelerated tumor progression, highlighting the complexity and potential risks of indiscriminate CAF targeting. In recent years, emerging therapies such as radionuclide therapy, CAR‐T cell therapy, and tumor vaccines directed at CAFs have yielded promising preclinical outcomes. Nonetheless, identifying specific markers for selective CAF phenotype elimination remains a significant challenge. Among the available approaches, CAF reprogramming has garnered particular attention. Treatment with ATRA has shown efficacy in restoring PSCs to a quiescent state in preclinical models of pancreatic cancer. However, the applicability of this strategy to other tumor types remains uncertain. As research into the molecular mechanisms of CAF activation progresses across various cancers, identifying key regulatory pathways and therapeutic targets for CAF reprogramming holds substantial potential for future treatment development. In recent years, the combination of CAF‐targeted therapies with conventional cancer treatment modalities, such as immunotherapy, chemotherapy, and radiotherapy, has been extensively investigated. Rather than eliminating specific CAF subtypes with ambiguous protumor or antitumor roles, integrating CAF‐targeted strategies with direct tumor cell cytotoxicity may yield more effective therapeutic outcomes. Furthermore, advances in targeted drug delivery systems have enabled more precise delivery of therapeutics to CAFs within the TME, thereby reducing off‐target effects and enhancing treatment specificity.

In addition to therapeutic strategies, diagnostic approaches targeting CAFs have garnered increasing attention, particularly those focusing on the specific marker FAP. Due to its widespread and persistent expression in various tumors, FAP has emerged as a key target in multimodal imaging. Among FAP‐targeted tracers, FAPI compounds have become the standard for both PET and fluorescence imaging because of their favorable pharmacokinetics and sustained clinical performance. In recent years, several novel tracers have been developed that show promise as potential next‐generation FAP‐targeting agents. Compared with conventional FDG–PET, FAPI‐based imaging offers a higher TBR and fewer false positives. Some novel FAPI probes, such as FAPI‐46, have been designed to enhance FAP targeting by reducing molecular weight, which improves tumor penetration. However, this modification also accelerates systemic clearance, potentially limiting tumor retention time. Consequently, the overall clinical efficacy of these probes remains to be fully established and warrants further investigation through larger clinical studies. Therefore, larger clinical trials are needed to validate their reliability and reproducibility across diverse patient populations. Fluorescence imaging, often utilized in intraoperative settings, benefits from FAP's strong stromal localization, allowing surgeons to more precisely delineate tumor margins. However, it is important to recognize the limitations of fluorescence imaging, particularly its susceptibility to off‐target effects and limited tissue penetration. One potential solution is the use of dual‐modal probes that combine PET and fluorescence imaging, which can not only improve diagnostic accuracy but also assist in identifying patient populations most likely to benefit from such approaches. Concurrently, structural optimization of these probes, such as enhancing photostability, reducing background signal, and improving tumor specificity, may further enhance their performance and clinical applicability. Continued efforts toward the clinical translation of FAP‐targeted fluorescence imaging are warranted, given its potential to improve surgical outcomes.

Ultimately, the heterogeneity of CAFs, both across different tumor types and among functional subgroups within the same tumor, contributes to a range of pathogenic mechanisms. This complexity presents opportunities for the development of targeted therapies aimed at key molecular drivers. Concurrently, with the advancement of precision medicine in oncology, identifying patient populations that may benefit from CAF‐targeted therapies has become increasingly important. Future research may focus on integrating CAF‐targeted imaging modalities to stratify potential responders. Achieving this goal will require close interdisciplinary collaboration among radiology, nuclear medicine, surgery, and oncology to establish a comprehensive framework for CAF‐specific diagnosis and treatment. The implementation of a theranostic approach, which combines diagnostic imaging and targeted therapy, is essential for guiding future investigations and ultimately translating CAF‐targeted strategies into clinically beneficial outcomes.

## Author Contributions

Y.L. and K.C. proposed this theme, organized the structure, provided the raw materials, and selected the cited references. Y.L., Q.L., X.J., X.Y., X.J., and Y.W. reorganized the figures and wrote the manuscript. K.C. revised the manuscript and oversaw the project. All authors reviewed the manuscript. All authors have read and approved the final version.

## Ethics Statement

The authors have nothing to report.

## Conflicts of Interest

The authors declare no conflicts of interest.

## Data Availability

The authors have nothing to report.
